# Examining SNP-SNP interactions and risk of clinical outcomes in colorectal cancer using multifactor dimensionality reduction based methods

**DOI:** 10.3389/fgene.2022.902217

**Published:** 2022-08-03

**Authors:** Aaron Curtis, Yajun Yu, Megan Carey, Patrick Parfrey, Yildiz E. Yilmaz, Sevtap Savas

**Affiliations:** ^1^ Discipline of Genetics, Faculty of Medicine, Memorial University, St. John’s, NL, Canada; ^2^ Division of Biomedical Sciences, Faculty of Medicine, Memorial University, St. John’s, NL, Canada; ^3^ Discipline of Medicine, Faculty of Medicine, Memorial University, St. John’s, NL, Canada; ^4^ Department of Mathematics and Statistics, Faculty of Science, Memorial University, St. John’s, NL, Canada; ^5^ Discipline of Oncology, Faculty of Medicine, Memorial University, St. John’s, NL, Canada

**Keywords:** colorectal cancer, MDR, MMPs, prognostic markers, protein interaction network, SNP-SNP interactions, VEGF family

## Abstract

**Background:** SNP interactions may explain the variable outcome risk among colorectal cancer patients. Examining SNP interactions is challenging, especially with large datasets. Multifactor Dimensionality Reduction (MDR)-based programs may address this problem.

**Objectives:** 1) To compare two MDR-based programs for their utility; and 2) to apply these programs to sets of MMP and VEGF-family gene SNPs in order to examine their interactions in relation to colorectal cancer survival outcomes.

**Methods:** This study applied two data reduction methods, Cox-MDR and GMDR 0.9, to study one to three way SNP interactions. Both programs were run using a 5-fold cross validation step and the top models were verified by permutation testing. Prognostic associations of the SNP interactions were verified using multivariable regression methods. Eight datasets, including SNPs from MMP family genes (*n* = 201) and seven sets of VEGF-family interaction networks (*n* = 1,517 SNPs) were examined.

**Results:** ∼90 million potential interactions were examined. Analyses in the MMP and VEGF gene family datasets found several novel 1- to 3-way SNP interactions. These interactions were able to distinguish between the patients with different outcome risks (regression *p*-values 0.03–2.2E-09). The strongest association was detected for a 3-way interaction including *CHRM3*.rs665159_*EPN1*.rs6509955_*PTGER3*.rs1327460 variants.

**Conclusion:** Our work demonstrates the utility of data reduction methods while identifying potential prognostic markers in colorectal cancer.

## Background

Colorectal cancer is a common disease accounting for ∼10% of the global cancer cases (Bray et al., 2018). The first years following diagnosis are critical and associated with a higher risk of negative disease outcomes (Yu et al., 2019). Select disease, tumor, and patient characteristics (Compton et al., 2000; Berian et al., 2015; Steele et al., 2019) are helpful while estimating prognosis and making treatment recommendations. Sadly, the survival rates vary across different countries and a significant portion of the patients are lost to this disease (5-years survival rate ∼<60%) (Coleman et al., 2008; Pathy et al., 2012; Arnold et al., 2017). In the current era of Personalized Medicine, one of the main aims is to identify additional prognostic markers that can help with better risk classification and improve patient outcomes.

Genetic variants, such as Single Nucleotide Polymorphisms (SNPs), are widely studied in prognostic research in oncology (Savas and Liu, 2009; Xu et al., 2015; Ziv et al., 2015). A common goal of this research area is to assess whether genetic variants are associated with, and hence, can be a marker of patient outcome risk. Survival studies examining genetic variants in colorectal cancer, including large-scale association studies (Pander et al., 2015; Xu et al., 2015; Phipps et al., 2016; Penney K. L. et al., 2019, Penney et al., 2019 M. E.; Yu et al., 2021) have mostly focused on analysis of SNPs one by one, assuming their individual effects and/or associations with the outcomes. This approach, while quite valuable, has also an obvious limitation: it misses detection of potential interactions among the variants.

It is possible that genetic variations jointly, but not alone, affect patient survival outcomes (i.e. interactions). That means that the effects of variants/genotypes are only detectable when they exist together in the patient genomes and are examined using specific approaches. While it is possible to examine interactions using statistical methods, these analyses may suffer from several well-known complexities (e.g. sparse data, need for computational resources), especially as the number of variables examined increases (Motsinger and Ritchie, 2006a). As an example of this complexity, the number of possible combinations of three SNPs, or “3-way interactions,” in a dataset of 100 SNPs is 161700, a large number of variables to study. Because of such methodological restrictions and the fact that there are large numbers of genetic variations in the human genome, it is necessary to apply other approaches, such as data reduction methods, for comprehensive SNP interaction analyses. Multifactor Dimensionality Reduction (MDR) is a data reduction method designed for use in studies examining the interactions among variables while accounting for difficulties inherent in interaction analysis (Ritchie et al., 2001). Initially created to support a small number of study designs, MDR has since been adapted for other types of studies. Generalized MDR (GMDR) (Lou et al., 2007) is an extension of MDR to support generalized linear models (e.g. logistic regression). Cox-MDR (Lee et al., 2012) is a type of GMDR which is designed specifically for survival/time-to-event studies and utilizes the Cox-regression method.

Studies that have so far considered the interactions of genetic variants in colorectal cancer outcomes using MDR are quite limited (Iglesias et al., 2009; Afzal et al., 2011a; Pander et al., 2011; Sarac et al., 2012; Hu et al., 2018; Jung et al., 2020). As a result, potential SNP interactions that may be associated with patient outcomes largely remain unknown. In this study, we aimed to explore the potential roles of SNP interactions in outcome risk of colorectal cancer patients using MDR-based methods. For this purpose, we utilized the genotype and outcome data of a cohort of colorectal cancer patients from Newfoundland and Labrador. We explored and compared the functionality of two MDR-based software—Cox-MDR (Lee et al., 2012) and GMDR 0.9 (Lou et al., 2007), and applied these software to examine the interactions among SNPs from the Matrix Metalloproteinase (MMP) family of genes and Vascular Endothelial Growth Factor (VEGF)-family interaction network genes. Our results show that there are unique limitations and strengths of Cox-MDR and GMDR 0.9, which should be considered in future studies. More importantly, our results identified novel SNP interactions that can help distinguish between colorectal cancer patients with significantly different outcome risks.

## Data and methods

### Ethics approval

This study was conducted with ethics approval by the Health Research Ethics Authority of Newfoundland and Labrador (HREB #2018.051; #2009.106). This study was a secondary use of data study, hence, HREB waived the requirement for patient consent.

Part 1: Exploration of Cox-MDR and GMDR 0.9 programs and analysis of interactions between the SNPs from the MMP family of genes.

### Patient cohort, genes selected, outcome measures, covariates, and data considerations

This is a cohort study. The baseline characteristics of the patient cohort included in this part of the study (*n* = 439) are shown in Supplementary Table S1. Patients were recruited by the Newfoundland Familial Colorectal Cancer Registry (NFCCR) (Green et al., 2007; Woods et al., 2010). They were under the age of 76 at the time of diagnosis and were diagnosed with colorectal cancer between 1999 and 2003. Pathological/clinical and follow-up data were collected from resources such as clinical reports, the Newfoundland Cancer Treatment and Research Foundation database, and follow-up questionnaires (Green et al., 2007; Woods et al., 2010; Negandhi et al., 2013; Yu et al., 2019). The date of last follow up was 2010. Genetic data was previously obtained from blood samples via the Illumina Omni1-Quad human SNP genotyping platform (reactions were outsourced to Centrillion Biosciences, United States), and sample quality control (QC) measures were implemented (Xu et al., 2015). As a result, all patients included into the analyses were of Caucasian ancestry and unrelated to each other (Xu et al., 2015).

Since one of our aims in Part 1 was to examine and compare the performance and functionality of the two MDR-based programs, we opted for a set of genes and SNPs that were previously examined in our lab (Supplementary Table S2). Specifically, the best suited genetic model for SNPs from the MMP genes and their one-by-one associations with patient outcomes were previously examined (Dan et al., 2016). This previous knowledge enabled us to assess the results of the 1-way interaction analyses obtained using the MDR methods during the current study. We kept the covariates and outcome measure examined in Part 1 the same as in that previous study. The covariates included age at diagnosis, disease stage, MSI (microsatellite instability)-status, and tumor location (rectum, colon). The outcome of interest was death from any cause (Overall Survival; OS).

Since Cox-MDR and GMDR 0.9 make their calculations, classify the patient genotypes as high-risk or low-risk, and select best models based on different scoring methods (i.e. martingale residuals obtained by Cox regression in Cox-MDR and logit score obtained by logistic regression in GMDR 0.9), Cox-MDR and GMDR 0.9 differ in data requirements. For example, as GMDR 0.9 utilizes logistic regression method, the 5-years-survival outcome measure was used. In Cox-MDR analysis, survival status and time to death (or the last date of alive contact) were used. Considering these and additional input data requirements for each program, a number of measures were taken while preparing the data files for analysis (see Supplementary Material for details). Since we aimed to compare their performance in this first part of the study, we also examined the same set of patients in the Cox-MDR and GMDR 0.9 analyses.

### Single Nucleotide Polymorphism genotype data and quality control measures

SNPs from the MMP family genes were extracted from the genome-wide SNP genotype data files using the gene genomic location information and the PLINK software (Purcell et al., 2007; PLINK, 2017 version 1.07), with the following quality control parameters being implemented: minor allele frequency (MAF) ≥ 0.05, Hardy-Weinberg Equilibrium (HWE) *p* > 0.0001, and missing genotype rate = 0. Pairwise squared correlation coefficient (*r* ^2^) values and MAFs were calculated using PLINK. When there were multiple SNPs with *r* ^2^ = 1 (i.e. those which would score identically using the MDR procedure), SNPs were removed such that only one of these SNPs was present in the final dataset. As a result, 201 SNPs from 21 MMP genes were included into the analysis (Supplementary Table S2).

### Cox-MDR and GMDR 0.9 analyses

**FIGURE 1:**
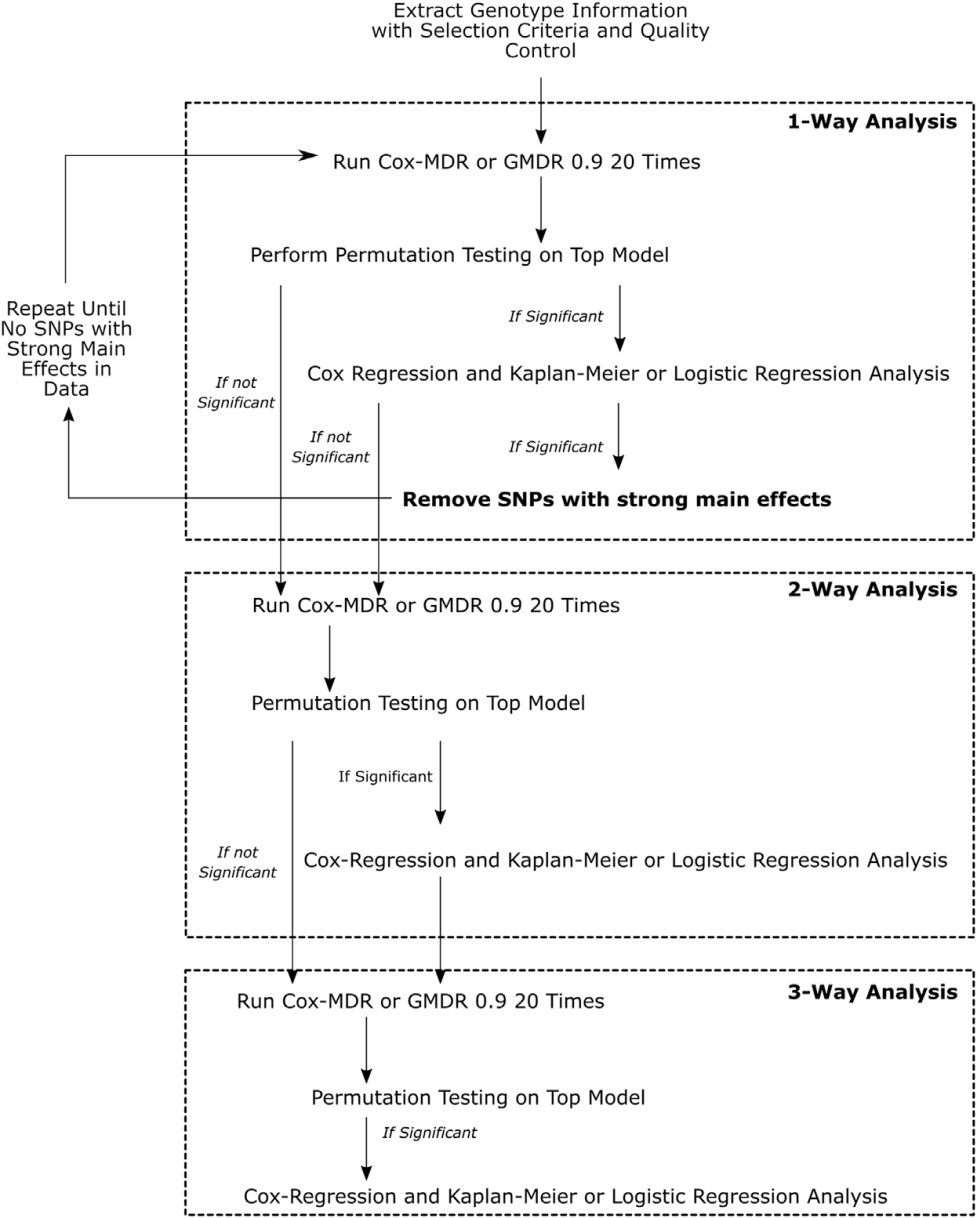
This figure demonstrates the overall workflow of the analyses performed. Multivariate Cox-regression and univariate Kaplan-Meier analyses were used to verify the Cox-MDR results and assess the associations of the identified genotype groups with clinical outcomes, whereas multivariate logistic regression was used to verify the GMDR 0.9 results and the association of the identified genotype groups with clinical outcomes.

The work-flow is summarized in Figure 1.

We focused on 1-way, 2-way, and 3-way (k = 1–3) interactions. 1-way interaction analysis examines whether the genotype groups of a single SNP may be categorized as high-risk and low-risk genotypes, and associated with an outcome/response variable. 2-way and 3-way interaction analyses examine whether combinations of genotype groups of two or three SNPs may be categorized as high-risk and low-risk genotypes, and associated with an outcome/response variable, respectively. Cox-MDR uses martingale residuals of Cox-regression models (Lee et al., 2012) and GMDR 0.9 (Lou et al., 2007) uses logit scores to categorize patient genotypes as high-risk and low-risk genotypes.

Cox-MDR code (Lee et al., 2012) was requested and received from the developer, Dr. Seungyeoun Lee (Sejong University, South Korea). We extended the code in order to add additional functionality and return the output that would be needed for our study using R (Core Team, 2017) (Supplementary Material). GMDR 0.9 code was downloaded from the UAB Department of Biostatistics Section on Statistical Genetics website (GMDR) on 11 December 2018. Command line arguments to set the random seeds were added to the permutation testing Perl script included with GMDR 0.9 (Supplementary Material). Once we verified that Cox-MDR worked as expected, it was run with the dataset (including both the clinical [i.e. covariates and OS time and status] and the genotype data of the SNPs from the MMP genes).

All interaction analyses were performed using a 5-fold cross-validation procedure. 5-fold cross validation is appropriate when the sample size is modest, like ours, while still providing adequate power (Motsinger and Ritchie, 2006b). 4/5 of these folds served as a training set for the MDR procedure and the final 1/5 was an independent testing set from which the final model score was derived. The code was run 20 times, each run yielding a “best Cox-MDR model,” with different random seeds to ensure different partitioning of the dataset into each of the five cross-validation folds (i.e. to reduce the influence of any specific partitioning of the data). Given the 5-fold cross-validation procedure, this resulted in each SNP or SNP combination being examined in potentially a total of 100 patient datasets. Among the best Cox-MDR models returned by each of the 20 runs, we prioritized the most frequently detected best Cox-MDR model (with consistent SNP ID(s) and high-risk and low risk genotype information) with the highest testing balance accuracy (TBA) score. We refer to these models as the “top” Cox-MDR models throughout this manuscript.

GMDR 0.9 was applied to the same dataset as used in Cox-MDR, with the only exception of using the 5-years survival status as the response variable. In contrast to Cox-MDR, GMDR 0.9 can only select the best models based on the cross-validation consistency (CVC); that is, the model with the highest CVC among cross-validation folds is selected. After running the GMDR 0.9 analysis 20 times, we selected the top model as in Cox-MDR and based on the highest average TBA value among cross validation folds (GMDR 0.9’s analogue to Cox-MDR’s highest TBA). In cases when there were multiple models satisfying the best MDR model criteria in a dataset, we used the TBA, and if still needed, the CVC information, as the tie breaker.

### Permutation testing

Once the top Cox-MDR or GMDR 0.9 model was identified, the significance of the model was assessed using permutation testing. For GMDR 0.9, permutation testing was performed using the included Perl script, which was extended to allow setting of random seeds. For Cox-MDR permutation testing, an R function was written. The permutation procedure was performed using 1,000 permutations of the data (Supplementary Material).

Permutation testing was performed for all top models selected from k-way runs (1-3-ways). As noted by others (Ritchie et al., 2001; Motsinger and Ritchie, 2006b; Edwards et al., 2009; Gui et al., 2011; Lee et al., 2012; De et al., 2015; Gola et al., 2016), it is possible that a single SNP with a strong main effect (that can be identified as the top MDR-model in 1-way analysis), may impact higher order interaction analysis when using MDR-based methods, and hence, needs to be removed from the 2-way and 3-way interaction analyses. Therefore, we first performed the permutation testing for the top MDR model identified in the 1-way analysis and, if it turned out to be a significant MDR model, then we assessed whether the high-risk and low-risk genotype groups of this top model were associated with survival outcomes in the patient cohort using statistical methods (see below). In the case where a significant association was detected, we then performed subsequent runs by excluding this SNP and any other SNP in the dataset that was in high linkage disequilibrium (LD) with it (*r* ^2^ ≥ 0.8). This SNP removal procedure was repeated until all SNPs with strong main effects in 1-way analyses were removed from the dataset (Figure 1). We then proceeded to 2-way and 3-way analyses on the final dataset with all SNPs with strong main effects removed.

### Kaplan-Meier curves and multivariable regression analyses

Following identification of a significant top MDR model by permutation testing, we assessed whether the high-risk and low-risk genotype groups of the model were associated with survival outcomes in the patient cohort. For this purpose, we applied multivariable Cox regression analysis (for the models identified by Cox-MDR) and logistic regression analysis (for the models identified by GMDR 0.9) using the same clinical covariates for adjustment that were used in the Cox-MDR and GMDR 0.9 runs. When needed, Kaplan-Meier curves were constructed to visualize the survival times of the patient groups with the high-risk and low-risk genotype groups over time. These analyses were performed using IBM SPSS Statistics software (versions 25 and 26, Armong, NY) (IBM SPSS Statistics for Windows, 2017) or R. A *p*-value of <0.05 was considered significant.

Part 2: Interactions among the SNPs of the VEGF interaction networks

Data resources and methods for Part 2 of this study were similar to Part 1, except for the differences outlined in this section. Four hundred patients (Supplementary Table S3) met the data requirements. All 400 of these patients were used in the Cox-MDR analysis. For Cox-MDR analysis, Disease Specific Survival (DSS) was used as an outcome measure, where the endpoint was death from colorectal cancer. For GMDR 0.9 analysis 5-years DSS time was used as the outcome measure. Using this outcome measure, five patients, who were censored prior to 5 years were excluded from analysis, as the survival status of these patients at 5 years was unknown. This left 395 patients for analysis with the GMDR 0.9 algorithm. An updated outcome data [with the last follow-up date of 2018 (Yu et al., 2019)] was used in this part of the study. Clinical variables that were previously identified as prognostic markers for DSS (Yu et al., 2019) were used as covariates in Cox-MDR, GMDR 0.9, and Cox regression and logistic regression analyses (tumor location, stage, MSI status, adjuvant chemotherapy, and radiotherapy status).

For this part of the study, we focused on the VEGF family members and examining SNP interactions in their protein-protein interaction networks. Four ligands (*VEGFA*, *VEGFB*, *VEGFC*, and *PIGF*) and three receptors (*VEGFR1*, *VEGFR2*, and *VEGFR3*) were selected. Since association studies using the sex chromosome genetic variations face additional complexities, the fifth ligand, *VEGFD*, which is located on the X chromosome, was not included.

### Identification of interaction partners of the VEGF family proteins

Each of the seven VEGF proteins were searched in the BioGRID 3.5 database (Stark et al., 2006; Oughtred et al., 2021; BioGRID | Database of protein, chemical, and genetic interactions) to find proteins that interact with them (i.e. protein-protein interaction networks; BioGRID accessed on 22 October 2019). Genomic locations for all interactors were obtained from the Ensembl database (Howe et al., 2021; Ensembl Genome Browser) using the legacy archive Biomart (Archives). PLINK was used for genotype extraction from the genome-wide SNP genotype data files, followed by LD-based pruning. Interactors located on the X chromosome (*FIGF*, *IKBKG*, and *VSIG4*) and genes with no SNPs after quality control and pruning steps (*BCS1L*, *CTGF*, *LRFN3*, *NUDT16L1*, *SCH1*, *TXNIP*, and *UBIAD1*) were excluded. In 7 VEGF networks, there was a total of 1,517 unique SNPs (number of SNPs in each set: *VEGFA* = 401; *VEGFB* = 174; *VEGFC* = 38; *PIGF* = 102; *VEGFR1* = 222; *VEGFR2* = 747; *VEGFR3* = 328) in a total of 131 unique genes (number of genes in each set: *VEGFA* = 43; *VEGFB* = 14; *VEGFC* = 3; *PIGF* = 5; *VEGFR1* = 15; *VEGFR2* = 68; *VEGFR3* = 23). Please see Supplementary Figure S1 and Supplementary Tables S4, S5 for the interaction networks, proteins in each interactome, and the IDs of SNPs retrieved and analyzed in this part of the study.

### Bioinformatics analyses

In order to explore the links between the SNPs of interest and clinical outcomes, we utilized literature reports (from PUBMED), and dbANGIO (Savas, 2012) and dbCPCO (Savas and Younghusband, 2010) databases. We also searched RegulomeDB (Boyle et al., 2012; RegulomeDB) and GTEx databases (Lonsdale et al., 2013) to identify eQTLs that are associated with expression levels of genes (Note that GTEx has no data for rectal tissues, so only transverse and sigmoid colon tissue information was available). Information on the type of variation (e.g. intronic) were retrieved from dbSNP (Sherry et al., 2001).

## Results

Part 1: Examination of the interactions between the MMP gene family SNPs using Cox-MDR and GMDR 0.9

Interactions among 201 SNPs from 21 MMP genes were examined as a set (a total of 1,353,601 potential interactions). As a result, 1-way Cox-MDR interaction analysis identified *MMP27*-rs11225388 (MAF = 0.27; an intronic SNP) and classified its genotypes as high-risk (AA) and low-risk (AG and GG) in the top MDR model. Permutation testing was also significant (*p* = 0.011). It is interesting that the best MDR-models identified by each of the 20 individual runs identified this SNP and its genotype categories consistently (Supplementary Table S6). Multivariable Cox regression analysis, adjusting the rs11225388 genotypes (low risk genotypes versus high risk) for clinical covariates, showed that this SNP genotype model was independently associated with OS (Table 1). Therefore, Cox-MDR successfully identified a significant 1-way interaction. These results also meant that the rs11225388 SNP had a significant main effect, which necessitated it (as well as two other SNPs with high LD with it: rs11225389 and rs12365082) being removed from the dataset prior to future analyses. Upon re-running Cox-MDR 1-way analysis and applying permutation testing to the top model, we did not identify a significant 1-way MDR model. We, therefore, proceeded with 2-way and 3-way analysis. These runs did not identify any significant multi-loci Cox-MDR models in this dataset.

**TABLE 1:** Multivariable Cox regression analysis result for the significant 1-way Cox-MDR model in the MMP dataset (overall survival).

Top model SNP	High risk genotypes	*p*-value	HR	95% CI (lower-upper)
rs11225388_GA	AA	0.002	0.591	0.425–0.821

CI: confidence interval; HR: hazards ratio; SNP: single nucleotide polymorphism. HR calculated for low risk genotypes (GG + GA) versus high-risk genotype (AA).

In contrast, in the 1-way analysis, GMDR 0.9 selection procedure did not identify a significant model following permutation testing. However, 2-way analysis identified a two-loci MDR model including the *MMP16*.rs7817382 and *MMP24*.rs2254207 variants (permutation testing *p* = 0.001; Table 2). Multivariable logistic regression analysis verified that this model had a significant association with 5-years survival of patients when adjusted for other prognostic covariates (high risk genotypes versus low risk genotypes; OR: 3.27; *p* = 4E-6). Both of these SNPs are non-coding region SNPs and were common in the patient cohort (MAFs = 0.25 and 0.26, respectively). Additionally, in the 3-way analysis, a GMDR 0.9 model including genotypes of *MMP16*.rs2664369, *MMP20*.rs11225332, and *MMP2*.rs11639960 variants were identified in the top model (permutation testing *p* < 0.001). Multivariable logistic regression analysis showed that this model distinguished patients based on their 5-years survival status independent of other covariates and this association was quite strong (*p* = 1.3E-8; OR: 4.5; Table 2). Kaplan Meier curves for the identified high-risk and low-risk genotypes are shown in Supplementary Figure S2. Rs2664369 is a 3′-untranslated region variant, and rs11225332 and rs11639960 are both intronic variants. These SNPs were common in the patient cohort (MAF = 0.43, 0.40, and 0.35, respectively).

Part 2: Examination of the interactions in the VEGF interaction network datasets using Cox-MDR and GMDR 0.9

**TABLE 2:** Multivariable logistic regression analysis results for the significant 2-way and 3-way GMDR 0.9 models in the MMP dataset (overall survival).

Top model SNPs	High risk genotypes	*p*-value	OR	95% CI (lower-upper)
rs7817382_GA and rs2254207_CA	(0AA,1CA), (0AA,2CC), (1GA,0AA), (1GA,2CC), (2GG,1CA)	4.4194E-06	3.266	1.971–5.414
rs2664369_GT, rs11225332_CT and rs11639960_GA	(0TT,0TT,2GG), (0TT,1CT,1GA), (0TT,1CT,2GG), (0TT,2CC,1GA), (1GT,0TT,0AA), (1GT,0TT,1GA), (1GT,1CT,2GG), (1GT,2CC,2GG), (2GG,0TT,0AA), (2GG,1CT,2GG), (2GG,2CC,0AA), (2GG,2CC,2GG)	1.2929E-08	4.503	2.681–7.563

CI: confidence interval; OR: odds ratio; SNP: single nucleotide polymorphism.

Alleles are given in the order major allele minor allele. 0,1,2 refer to additive coding, i.e. dosage of the minor allele (0 = 0 copies of the minor allele, 1 = 1 copies of the minor allele, 2 = 2 copies of the minor allele).

In this part of the study, we investigated SNP interactions separately for seven sets of VEGF family protein interaction networks (Supplementary Tables S4, S5). Altogether, these analyses examined 88,989,448 potential interactions.

**FIGURE 2:**
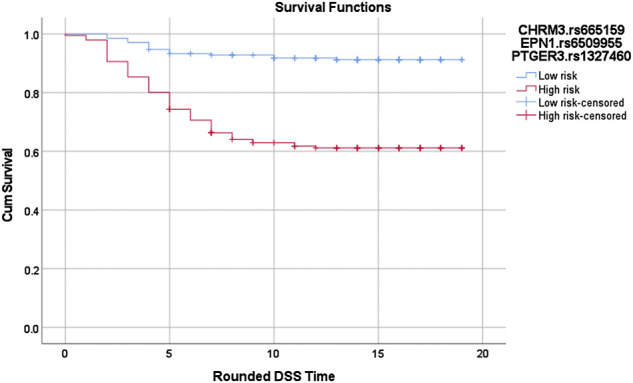
Log-rank *p* = 1.02619688760668E-12. Red: high risk genotype combinations: (TC,GG,GG), (CC,AG,GG), (TC,AG,GG), (CC,AA,GG), (CC,GG,AG), (CC,AG,AG), (CC,AA,AG), (TC,AA,AG), (TC,GG,AA), (TT,GG,AA), (TC,AG,AA), (CC,AA,AA), and (TT,AA,AA). Blue: all other genotype combinations. The vertical lines on the curves denote the censored patients (e.g. patients alive at the last follow up time). *X* and *Y* axis show the follow-up time (in years; rounded) and cumulative survival, respectively.

Cox-MDR identified four significant MDR models, three of which were also confirmed by multivariable Cox regression analysis (Table 3). In the 1-way analysis of the *PIGF* network, we identified one SNP associated with DSS (*RNF123*.rs11130216). Additionally, both 2-way and 3-way interactions were detected and they were both identified during the *VEGFR3* network analysis. These multi-loci interactions include SNPs from *CHRM3*, *PTGER3*, or *EPN1* genes. The strongest association with disease-specific survival was detected in the 3-way analysis with a very strong *p*-value of 2.21E-09 (*CHRM3*.rs665159_*EPN1*.rs6509955_*PTGER3*.rs1327460; HR: 5.0). As also demonstrated by the Kaplan Meier curve (Figure 2), this model’s genotype classification was able to clearly separate patients based on their outcome risks.

**TABLE 3:** Permutation testing and multivariable Cox-regression analysis results for the top Cox-MDR models in the VEGF interaction network set analyses (disease specific survival).

Interactor set	Top model SNP(s)	High risk genotypes	Permutation *p*-value	Cox regression *p*-value	HR	95% CI (lower-upper)
1-way						
*Iteration 1*						
*VEGFA*	*FN1*.rs2289200 [TG]	1 (TG),2 (TT)	0.273	**—**	**—**	**—**
*VEGFB*	*VEGFA*.rs833070 [GA]	1 (GA)	0.201	**—**	**—**	**—**
*VEGFC*	*VEGFC*.rs1485766 [CA]	1 (CA)	0.346	**—**	**—**	**—**
*VEGFR1*	*PIK3R1*.rs4122269 [CT]	0 (TT)	0.07	**—**	**—**	**—**
*VEGFR2*	*PTPN12*.rs1024723 [TC]	0 (CC),2 (TT)	0.181	**—**	**—**	**—**
*VEGFR3*	*LRRK1*.rs930847 [CA]	1 (CA),2 (CC)	0.098	**—**	**—**	**—**
*PIGF*	*RNF123*.rs11130216 [AC]	1 (AC),2 (AA)	0.032	0.003	1.977	1.265–3.089
*Iteration 2*						
*PIGF*	*VEGFA*.rs833070 [GA]	1 (GA)	0.045	0.298	1.256	0.818–1.928
2-way						
*VEGFA*	*CLU*.rs7982 [TC], *FLT1*.rs7332329 [GA]	(0 [CC],0 [AA]), (1 [TC],1 [GA]) (0 [CC],2 [GG]) (2 [TT],2 [GG])	0.392	**—**	**—**	**—**
*VEGFB*	*FAT1*.rs10155467 [TC], *VEGFA*.rs3025010 [CT]	(1 [TC],0 [TT]) (0 [CC],1 [CT]) (2 [TT],1 [CT]) (0 [CC],2 [CC]) (2 [TT],2 [CC])	0.225	**—**	**—**	**—**
*VEGFC*	*KDR*.rs17709898 [GA], *VEGFC*.rs3775195 [AC]	(0 [AA],0 [CC]) (2 [GG],0 [CC]) (1 [GA],1 [AC]) (0 [AA],2 [AA]) (1 [GA],2 [AA])	0.146	**—**	**—**	**—**
*VEGFR1*	*FLT1*.rs9551462 [TC], *PIK3R1*.rs1823023 [AG]	(1 [TC],0 [GG]) (2 [TT],0 [GG]) (0 [CC],1 [AG]) (0 [CC],2 [AA]) (1 [TC],2 [AA])	0.128	**—**	**—**	**—**
*VEGFR2*	*APP*.rs2096488 [CA], *DNM2*.rs7246673 [TG]	(2 [CC],0 [GG]) (0 [AA],1 [TG]) (1 [CA],2 [TT]) (2 [CC],2 [TT])	0.389	**—**	**—**	**—**
*VEGFR3*	*CHRM3*.rs665159 [TC], *PTGER3*.rs1327460 [AG]	(0 [CC],0 [GG]) (1 [TC],0 [GG]) (0 [CC],1 [AG]) (1 [TC],2 [AA])	0.004	2.03E-06	3.147	1.961–5.050
*PIGF*	*NRP1*.rs2474723 [GA], *RNF123*.kgp9864706 [AG]	(0 [AA],0 [GG]) (1 [GA],2 [AA])	0.527	**—**	**—**	**—**
3-way						
*VEGFA*	*FOS*.rs7101 [CT], *NRP2*.rs861079 [TC], *TFAP2A*.rs303055 [CT]	(0 [TT],0 [CC],0 [TT]) (1 [CT],0 [CC],0 [TT]) (0 [TT],1 [TC],0 [TT]) (0 [TT],2 [TT],0 [TT]) (0 [TT],0 [CC],1 [CT]) (1 [CT],1 [TC],1 [CT]) (2 [CC],1 [TC],1 [CT]) (0 [TT],2 [TT],1 [CT]) (1 [CT],2 [TT],1 [CT]) (2 [CC],2 [TT],1 [CT]) (0 [TT],0 [CC],2 [CC]) (2 [CC],0 [CC],2 [CC]) (1 [CT],2 [TT],2 [CC])	0.058	**—**	**—**	**—**
*VEGFB*	*ALOXE3*.rs3809882 [CA], *COL6A2*.rs7280485 [AG], *NRP1*.rs6481844 [CT]	(1 [CA],0 [GG],0 [TT]) (0 [AA],1 [AG],0 [TT]) (2 [CC],1 [AG],0 [TT]) (1 [CA],2 [AA],0 [TT]) (0 [AA],0 [GG],1 [CT]) (2 [CC],0 [GG],1 [CT]) (0 [AA],1 [AG],1 [CT]) (1 [CA],2 [AA],1 [CT]) (2 [CC],2 [AA],1 [CT]) (1 [CA],1 [AG],2 [CC]) (2 [CC],2 [AA],2 [CC])	0.217	**—**	**—**	**—**
*VEGFC*	*FLT4*.rs2242217 [CT], *FLT4*.rs11748431 [AG], *VEGFC*.rs1485762 [TC]	(2 [CC],0 [GG],0 [CC]) (0 [TT],1 [AG],0 [CC]) (2 [CC],1 [AG],0 [CC]) (1 [CT],2 [AA],0 [CC]) (1 [CT],0 [GG],1 [TC]) (2 [CC],0 [GG],1 [TC]) (1 [CT],1 [AG],1 [TC]) (2 [CC],1 [AG],1 [TC]) (1 [CT],2 [AA],1 [TC]) (0 [TT],0 [GG],2 [TT]) (2 [CC],0 [GG],2 [TT]) (1 [CT],1 [AG],2 [TT])	0.229	**--**	**--**	**--**
*VEGFR1*	*FLT1*.rs12429309 [CT], *FLT1*.rs9551462 [TC], *PIK3R1*.rs1823023 [AG]	(1 [CT],0 [CC],0 [GG]) (1 [CT],1 [TC],0 [GG]) (2 [CC],1 [TC],0 [GG]) (0 [TT],2 [TT],0 [GG]) (0 [TT],0 [CC],1 [AG]) (0 [TT],0 [CC],2 [AA]) (2 [CC],0 [CC],2 [AA]) (0 [TT],1 [TC],2 [AA]) (1 [CT],1 [TC],2 [AA])	0.097	**—**	**—**	**—**
*VEGFR2*	*COL18A1*.rs4819101 [AG], *NCOA4*.rs10761581 [GT], *PALLD*.rs10004025 [TC]	(0 [GG],0 [TT],0 [CC]) (1 [AG],0 [TT],0 [CC]) (2 [AA],0 [TT],0 [CC]) (0 [GG],1 [GT],0 [CC]) (2 [AA],0 [TT],1 [TC]) (0 [GG],1 [GT],1 [TC]) (0 [GG],2 [GG],1 [TC]) (1 [AG],2 [GG],1 [TC]) (1 [AG],0 [TT],2 [TT]) (2 [AA],0 [TT],2 [TT]) (1 [AG],2 [GG],2 [TT]) (2 [AA],2 [GG],2 [TT])	0.12	**—**	**—**	**—**
*VEGFR3*	*CHRM3*.rs665159 [TC], *EPN1*.rs6509955 [AG], *PTGER3*.rs1327460 [AG]	(1 [TC],0 [GG],0 [GG]) (0 [CC],1 [AG],0 [GG]) (1 [TC],1 [AG],0 [GG]) (0 [CC],2 [AA],0 [GG]) (0 [CC],0 [GG],1 [AG]) (0 [CC],1 [AG],1 [AG]) (0 [CC],2 [AA],1 [AG]) (1 [TC],2 [AA],1 [AG]) (1 [TC],0 [GG],2 [AA]) (2 [TT],0 [GG],2 [AA]) (1 [TC],1 [AG],2 [AA]) (0 [CC],2 [AA],2 [AA]) (2 [TT],2 [AA],2 [AA])	0.007	2.21E-09	5.004	2.952–8.481
*PIGF*	*FLT1*.rs17086609 [GA], *FLT1*.rs1853581 [CA], *NRP1*.rs2506141 [CT]	(1 [GA],0 [AA],0 [TT]) (0 [AA],1 [CA],0 [TT]) (0 [AA],2 [CC],0 [TT]) (2 [GG],2 [CC],0 [TT]) (1 [GA],0 [AA],1 [CT]) (0 [AA],1 [CA],1 [CT]) (2 [GG],1 [CA],1 [CT]) (0 [AA],0 [AA],2 [CC]) (2 [GG],0 [AA],2 [CC]) (2 [GG],1 [CA],2 [CC])	0.253	**—**	**—**	**—**

CI: confidence interval; HR: hazards ratio; SNP: single nucleotide polymorphism.

0, 1, and 2 in the High Risk Genotype column refer to additive coding, where the number refers to the number of minor alleles in the genotype.

Square brackets in the Top Model SNPs column indicate major and minor alleles for each SNP; which the first letter represents the minor allele and the second letter represents the major allele. In the high risk genotypes column, the three items enclosed in parentheses signify the genotypes of the combination of SNPs which was found to be high risk by Cox-MDR. Commas separate the genotypes for each SNP in the order in which they appear in the corresponding Top Model SNPs entry. Whenever a SNP with a main effect was identified in 1-way analysis, the analysis was repeated with that SNP removed from the dataset (i.e. successive iterations). *FLT1* is also known as *VEGFR1*; *KDR* is also known as *VEGFR2*; *FLT4* is also known as *VEGFR3*; and *PGF* is also known as *PIGF*.

**FIGURE 3:**
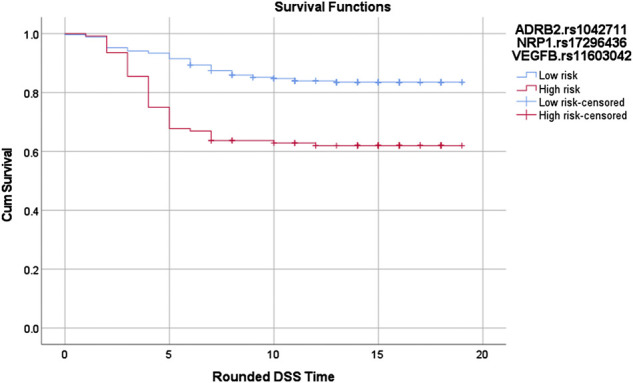
Log-rank *p* = 6.61897020900234E-07. Red: High risk genotypes: (TT,AA,TG), (TT,AA,TT), (TT,GA,TT), (TT,GG,TG), (CT,AA,TT), (CT,GA,GG), (CT,GG,GG), (CT,GG,TG), (CC,GA,GG), (CC,GA,TG), and (CC,GA,TT). Blue: All others except (CT,GG,TT) and (CC,GG,TT)The vertical lines on the curves denote the censored patients (e.g. patients alive at the last follow up time). X and Y axis show the follow-up time (in years; rounded) and cumulative survival, respectively.SNP-SNP interactions in survival outcomes.

Similar to Cox-MDR, GMDR 0.9 also identified interactions that were able to distinguish between patients with different outcome risk (the multivariable logistic regression *p*-values 0.032–2.4E-09; Table 4). GMDR 0.9 identified a larger number significant interactions than Cox-MDR (11, six, and seven 1-way, 2-way, and 3-way interactions, respectively). The strongest association with DSS (*p* = 2.4E-09) was detected for the 3-way *ADRB2*.rs1042711_*NRP1*.rs17296436_*VEGFB*.rs11603042 interaction in the *VEGFB* network analysis (HR: 10, 95% CI: 4.691–21.276; Kaplan Meier curves for the high-risk and low-risk genotypes are shown in Figure 3). Overall, the significant associations, particularly for multi-loci interactions, were quite encouraging. Generally, the significance levels of interactions increased with the order of interactions (i.e. from 1-way to 3-way). Of note, 3-way analysis identified significant interactions in all seven VEGF interaction networks examined. Rarely, interaction models included both the VEGF ligand and receptor (*FLT4*.rs307823_*KDR*.rs6828477_*KDR*.rs12502008) or two SNPs from the same gene (*FLT4*.rs11739750_*FLT4*.rs307814; Table 4), both detected in the *VEGFC* interaction network. For interested readers, the Kaplan Meier curves for the GMDR 0.9 identified interactions are shown in Supplementary Figure S3.

**TABLE 4:** Multivariable logistic regression analysis results for the top GMDR 0.9 models in the VEGF interaction network set analyses (disease-specific survival).

Interaction set	Top model SNP(s)	High risk genotypes	Permutation *p*-value	Logistic regression *p*-value	OR	95% CI (lower-upper)
1-way						
*Iteration 1*						
*VEGFA*	*NRP2*.rs3771003 [TG]	0 [GG],2 [TT]	0.014	0.010	2.399	1.230–4.679
*VEGFB*	*COL6A2*.rs9978018 [GA]	0 [AA],2 [GG]	0.02	0.032	2.015	1.062–3.822
*VEGFC*	*FLT4*.rs3797102 [CT]	1 [CT],2 [CC]	0.358	**—**	**—**	
*VEGFR1*	*MICAL2*.rs11022250 [GT]	0 [TT]	<0.001	0.002	2.941	1.468–5.891
*VEGFR2*	*PTPN12*.rs1024723 [TC]	0 [CC],2 [TT]	<0.001	1.442E-04	3.662	1.875–7.152
*VEGFR3*	*CHRM3*.rs12037424 [CT]	0 [TT]	0.005	0.004	2.616	1.369–4.997
*PIGF*	*RNF123*.rs11130216 [AC]	1 [AC],2 [AA]	0.045	0.011	2.359	1.222–4.554
*Iteration 2*						
*VEGFA*	*HNRNPL*.rs10403012 [GA]	0 [AA]	0.022	0.012	1.984	0.673–5.847
*VEGFB*	*VEGFB*.rs11603042 [TG]	1 [TG],2 [TT]	0.067	**—**	**—**	
*VEGFR1*	*MICAL2*.rs988189 [TC]	1 [TC],2 [TT]	0.116	**—**	**—**	
*VEGFR2*	*MAPK1*.rs2298432 [AC]	0 [CC]	0.001	3.425E-04	3.467	1.756–6.848
*VEGFR3*	*CHRM3*.rs2278642 [TG]	1 [TG],2 [TT]	0.007	0.006	2.924	1.362–6.278
*PIGF*	*FLT1*.rs3936415 [AG]	0 [GG]	0.069	**—**	**—**	
*Iteration 3*						
*VEGFA*	*HNRNPL*.rs2278012 [CT]	0 [TT]	0.051	**—**	**—**	
*VEGFR2*	*DNM2*.rs7246673 [TG]	1 [TG],2 [TT]	0.079	**—**	**—**	
*VEGFR3*	*LRRK1*.rs12595297 [GT]	1 [GT]	0.007	0.011	2.243	1.207–4.169
*Iteration 4*						
*VEGFR3*	*LRRK1*.rs17161155 [AG]	0 [GG]	0.043	0.009	2.317	1.235–4.346
*Iteration 5*						
*VEGFR3*	*CHRM3*.rs6692711 [TC]	1 [TC]	0.225	**—**	**—**	
2-way						
*VEGFA*	*ELAVL1*.rs3786619 [AG] *FLT1*.rs3936415 [AG]	(0 [GG],2 [AA]) (1 [AG],0 [GG]) (2 [AA],0 [GG]) (2 [AA],1 [AG])	<0.001	3.180E-05	4.387	2.186–8.805
*VEGFB*	*ADRB2*.rs1042711 [CT] *HAL*.rs3213737 [CT]	(0 [TT],1 [CT]) (1 [CT],0 [TT]) (2 [CC],1 [CT])	0.018	7.082E-05	3.696	1.940–7.044
*VEGFC*	*FLT4*.rs11739750 [TC] *FLT4*.rs307814 [TC]	(0 [CC],1 [TC]) (1 [TC],0 [CC]) (1 [TC],2 [TT]) (2 [TT],1 [TC])	0.002	1.335E-04	3.827	1.922–7.620
*VEGFR1*	*FLT1*.rs3794397 [TC] *MICAL2*.rs7946327 [CA]	(0 [CC],0 [AA]) (1 [TC],1 [CA]) (2 [TT],1 [CA])	0.003	1.852E-04	3.361	1.780–6.346
*VEGFR2*	*COL18A1*.rs7278425 [TC] *PTPRR*.rs4760847 [GA]	(0 [CC],1 [GA]) (1 [TC],0 [AA])	<0.001	1.213E-05	4.542	2.306–8.947
*VEGFR3*	*CHRM3*.rs1782357 [TC] *TMEM52B*.rs10505752 [TC]	(0 [CC],0 [CC]) (1 [TC],1 [TC]) (1 [TC],2 [TT]) (2 [TT],2 [TT])	<0.001	3.872E-05	3.892	2.037–7.433
*PIGF*	*FLT1*.rs2387632 [TC] *NRP1*.rs12762312 [TC]	(0 [CC],1 [TC]) (1 [TC],0 [CC]) (1 [TC],2 [TT]) (2 [TT],0 [CC])	0.055	**—**	**—**	
3-way						
*VEGFA*	*CLU*.rs9331888 [CG] *ELAVL1*.rs3786619 [AG] *NRP2*.rs861079 [TC]	(0 [GG],0 [GG],1 [TC]) (0 [GG],0 [GG],2 [TT]) (0 [GG],1 [AG],1 [TC]) (0 [GG],2 [AA],0[CC]) (0 [GG],2 [AA],2 [TT]) (1 [CG],1 [AG],0 [CC]) (1 [CG],1 [AG],2 [TT]) (1 [CG],2 [AA],0 [CC]) (2 [CC],0 [GG],1 [TC]) (2 [CC],0 [GG],2 [TT]) (2 [CC],1 [AG],2 [TT]) (2 [CC],2 [AA],2 [TT])	0.001	2.146E-07	9.322	4.010–21.672
*VEGFB*	*ADRB2*.rs1042711 [CT] *NRP1*.rs17296436 [GA] *VEGFB*.rs11603042 [TG]	(0 [TT],0 [AA],1 [TG]) (0 [TT],0 [AA],2 [TT]) (0 [TT],1 [GA],2 [TT]) (0 [TT],2 [GG],1 [TG]) (1 [CT],0 [AA],2 [TT]) (1 [CT],1 [GA],0 [GG]) (1 [CT],2 [GG],0 [GG]) (1 [CT],2 [GG],1 [TG]) (2 [CC],1 [GA],0 [GG]) (2 [CC],1 [GA],1 [TG]) (2 [CC],1 [GA],2 [TT])	0.007	2.404E-09	9.991	4.691–21.276
*VEGFC*	*FLT4*.rs307823 [GA] *KDR*.rs6828477 [CT] *KDR*.rs12502008 [TG]	(0 [AA],0 [TT],1 [TG]) (0 [AA],1 [CT],0 [GG]) (0 [AA],2 [CC],0 [GG]) (0 [AA],2 [CC],1 [TG]) (1 [GA],0 [TT],0 [GG]) (1 [GA],1 [CT],1 [TG]) (1 [GA],2 [CC],2 [TT]) (2 [GG],0 [TT],1 [TG]) (2 [GG],1 [CT],1 [TG]) (2 [GG],1 [CT],2 [TT])	0.038	4.028E-06	5.418	2.642–11.114
*VEGFR1*	*MICAL2*.rs1564947 [AG] *MICAL2*.rs954428 [GA] *NEDD4*.rs12232351 [AT]	(0 [GG],0 [AA],0 [TT]) (0 [GG],0 [AA],2 [AA]) (0 [GG],1 [GA],0 [TT]) (0 [GG],1 [GA],1 [AT]) (0 [GG],2 [GG],0 [TT]) (1 [AG],0 [AA],0 [TT]) (1 [AG],1 [GA],1 [AT]) (2 [AA],2 [GG],0 [TT]) (2 [AA],2 [GG],2 [AA])	<0.001	3.505E-08	14.855	5.693–38.761
*VEGFR2*	*DNM2*.rs7246673 [TG] *NRP1*.rs10827227 [TC] *SCUBE2*.rs7106593 [GT]	(1 [TG],0 [CC],1 [GT]) (1 [TG],1 [TC],0 [TT]) (1 [TG],1 [TC],2 [GG]) (1 [TG],2 [TT],0 [TT]) (1 [TG],2 [TT],1 [GT]) (1 [TG],2 [TT],2 [GG]) (2 [TT],0 [CC],2 [GG]) (2 [TT],1 [TC],1 [GT]) (2 [TT],1 [TC],2 [GG]) (2 [TT],2 [TT],1 [GT])	<0.001	7.062E-09	8.712	4.186–18.129
*VEGFR3*	*CHRM3*.rs1782357 [TC] *CHRM3*.rs685960 [CT] *TMEM52B*.rs10505752 [TC]	(0 [CC],0 [TT],0 [CC]) (0 [CC],1 [CT],0 [CC]) (1 [TC],0 [TT],1 [TC]) (1 [TC],0 [TT],2 [TT]) (1 [TC],1 [CT],0 [CC]) (2 [TT],0 [TT],2 [TT]) (2 [TT],1 [CT],0 [CC]) (2 [TT],1 [CT],2 [TT])	<0.001	5.721E-08	8.030	3.784–17.038
*PIGF*	*FLT1*.rs3936415 [AG] *FLT1*.rs11149523 [AG] *NRP1*.rs2073320 [TC]	(0 [GG],0 [GG],0 [CC]) (0 [GG],0 [GG],1 [TC]) (0 [GG],1 [AG],0 [CC]) (0 [GG],1 [AG],2 [TT]) (0 [GG],2 [AA],0 [CC]) (0 [GG],2 [AA],2 [TT]) (1 [AG],0 [GG],2 [TT]) (1 [AG],1 [AG],1 [TC]) (1 [AG],2 [AA],1 [TC]) (1 [AG],2 [AA],2 [TT]) (2 [AA],0 [GG],0 [CC]) (2 [AA],2 [AA],0 [CC])	<0.001	4.218E-07	12.996	4.812–35.103

CI: confidence interval; OR: odds ratio; SNP: single nucleotide polymorphism.

0, 1, and 2 in the High Risk Genotype column refer to additive coding, where the number refers to the number of minor alleles in the genotype.

Square brackets in the Top Model SNPs column indicate major and minor alleles for each SNP; in which the first letter represents the minor allele and the second letter represents the major allele. The High risk genotypes column lists genotypes which were found by GMDR 0.9 to be high risk for poor survival. High-risk genotypes have the following format: the items between each pair of parentheses specify a genotype which is high risk for poor survival according to the GMDR output, presented in the order of the SNPs listed in the Top model SNP column. e.g. for top model SNPs FLT1.rs3936415 [AG]_FLT1.rs11149523 [AG]_NRP1.rs2073320 [TC], genotypes (0 [GG],0 [GG],0 [CC]), rs3936415 = GG, rs11149523 = GG, and rs2073320 = CC were classified as high risk by the GMDR 0.9 procedure. Whenever a SNP with a main effect was identified in 1-way analysis, the analysis was repeated with that SNP removed from the dataset (i.e. successive iterations). *FLT1* is also known as *VEGFR1*; *KDR* is also known as *VEGFR2*; *FLT4* is also known as *VEGFR3*; and *PGF* is also known as *PIGF*.

### Comparison of Cox-MDR and GMDR 0.9 results

Both Cox-MDR and GMDR 0.9 identified *RNF123*.rs11130216 SNP in the 1-way analysis of the *PIGF* network. In both cases, the same genotypes were identified as high-risk and were associated with DSS in multivariable models. All other significant interactions were identified by either of the programs. Our results, hence, showed that there was little overlap between the results provided by Cox-MDR and GMDR 0.9. This may be initially attributed to the use of different scoring systems and response variables by these programs. However, Cox-MDR was the software which identified the *MMP27*.rs11225388 variant, as well as the high-risk/low-genotype classification, that was previously identified to be associated with OS in a highly similar patient cohort (Dan et al., 2016). Of note, this SNP had the strongest association in that dataset, so it is being identified by Cox-MDR and in all of the 20 1-way runs as the best SNP is quite striking (Supplementary Table S6). This SNP, however, was missed by GMDR 0.9. In addition, in GMDR 0.9, it was observed that there was no obvious way in which ties between “best models” (i.e. multiple “best models” with equal CVC values when selecting the best model) were being resolved. To test the effect of SNP order in the input data file, *MMP27*.rs11225388, a SNP with a known statistical association (see above), was moved to the beginning of the data file. This change resulted in significantly different GMDR 0.9 results (making rs11225388 the top SNP identified for this analysis) and thus, showed that input SNP order can affect results when the CVC is 1 or 2, out of a possible 5 (when multiple best models have the same CVC). Further observation confirmed that the earliest SNP in the dataset is chosen by GMDR 0.9 in the event of a CVC tie. Therefore, this not only explains why GMDR 0.9 missed this SNP, but also an important limitation of this and any other MDR software that uses CVC to pick the best model. Despite its limitation, it is worth noting that GMDR 0.9 also identified a number of models that were missed by Cox-MDR and distinguished patients based on their significantly different outcome risks (Tables 2, 4).

## Discussion

In this study, we explored the functionality and feasibility of two MDR-based programs, Cox-MDR (Lee et al., 2012) and GMDR 0.9 (Lou et al., 2007) and applied them to examine single-locus and multi-loci interactions in MMP family and VEGF interaction network genes in relation to survival outcome risks in colorectal cancer. Our results identified novel and statistically significant interactions that predicted the survival outcomes in colorectal cancer. Our results also showed that these two programs generally yielded different top MDR models and interactions, hence, they can be considered complementary while examining SNP interactions. To our knowledge, this is the first large-scale MDR analysis study that examined SNP interactions in relation to colorectal cancer outcomes.

Interactions among variables are understudied in cancer research. It is possible that the interactions among genetic variables, such as SNPs, play a role in survival outcomes biologically. Hence, limiting a study to associations of individual SNPs and survival outcomes has the potential to miss not only genetic relationships but also important biological information. In this regard, there has been little work done on studying multi-loci interactions in colorectal cancer with respect to survival outcomes, especially using a large number of variants. For example, limited MDR-based interaction analyses were conducted (Iglesias et al., 2009; Afzal et al., 2011a, 2011b; Pander et al., 2011; Sarac et al., 2012; Hu et al., 2018), investigating the interactions among a small number of polymorphisms (*n* = 5–17). These studies identified interacting polymorphisms that are associated with treatment response and/or survival outcomes. Therefore, while there has been little research on multi-loci interactions in colorectal cancer with respect to survival outcomes, there is also great potential in this area of research—this was our motivation to conduct this study. Additionally, in this study, we prioritized biologically relevant genes with well-known roles in disease progression in cancer: MMP family of genes and genes whose protein products were members of the protein interaction networks of seven separate VEGF-family proteins. Protein products of MMP family genes are involved in tissue remodeling, some of them have abnormalities associated with tumor invasion, tumor microenvironment, or metastasis (Hua et al., 2011). VEGF family of proteins are also involved in important cellular processes, and include VEGF ligands and receptors with roles in angiogenesis or lymphangiogenesis—two cellular mechanisms involved in tumor growth, invasion, and metastasis (Hicklin and Ellis, 2005; Lohela et al., 2009; Alitalo and Detmar, 2012). Therefore, the results of this study have the potential to provide new insights into the relationship of these genes, molecular pathways, and processes with the outcome risk in colorectal cancer.

In this study, we first verified whether the MDR-based methods are indeed useful in distinguishing genotypes as high-risk and low risk. In the analysis of the interactions among the MMP family gene SNPs, 1-way Cox-MDR analysis was in fact able to identify a SNP in the dataset which has a known main effect, i.e. associated with the OS in the patient cohort under dominant genetic model (*MMP27*.rs11225388). This SNP was previously examined in our lab using a similar patient cohort and using Cox regression method and it had the strongest association in the SNP set (Dan et al., 2016). This previous study had also shown the dominant genetic model as the best model explaining the relationship of the genotypes of this SNP with patient overall survival times. In the current study, association of *MMP27*.rs11225388 under the dominant genetic model with the OS times in the study cohort was also confirmed by Cox-MDR, classifying the high-risk and low risk genotypes correctly (Table 1). Therefore, Cox-MDR was able to identify a SNP significantly associated with the outcome measure and its genetic model correctly, which increased our confidence in Cox-MDR results, though Cox-MDR did not identify any multi-loci interactions in this data set.

In contrast, GMDR 0.9 identified two novel multi-loci interactions in the MMP dataset; *MMP16*.rs7817382_*MMP24*.rs2254207 and *MMP16*.rs2664369_*MMP20*.rs11225332_*MMP2*.rs11639960 (Table 2). Interestingly, both of the variants identified in 2-way analysis (*MMP16*.rs7817382 and *MMP24*.rs2254207) are also eQTLs and associated with the expression levels of *MMP16* and *MMP24-AS1* genes, respectively (Supplementary Table S7). Protein products of *MMP16* and *MMP24* are known to interact physically with pro-MMP2 and activate it by means of proteolytic cleavage (Llano et al., 1999; Zhao et al., 2004). *MMP2* has been linked to several human cancers, including colorectal cancer previously (van der Jagt et al., 2010; Dong et al., 2011; Wang et al., 2014; Ren et al., 2015; Gao et al., 2016; Jia et al., 2017). Therefore, it is possible that the role of both *MMP16* and *MMP24* in affecting the action of *MMP2* could explain the biology underlying the interaction identified by 2-way GMDR analysis. Additionally, one of the SNPs identified in the 3-way GMDR 0.9 analysis, *MMP2*.rs11639960, is an eQTL, affecting the expression levels of the gene called *LPCAT2*. *LPCAT2* is known to affect response to chemotherapy in colorectal cancer patients through an association with lipid droplet formation (Cotte et al., 2018). This SNP was also associated with prostate (Jacobs et al., 2008), and ovarian cancer risks (Velapasamy et al., 2013), as well as overall survival in colorectal cancer (Scherer et al., 2014). Two of the genes identified in 3-way GMDR analysis are known to be associated with colorectal cancer. As mentioned above, *MMP2* has been shown to be overexpressed in colorectal cancer tumors compared to normal tissues (Dong et al., 2011; Gao et al., 2016), and is associated with metastatic tumor phenotype (Dong et al., 2011; Gao et al., 2016) and shorter survival times in colorectal cancer (Dong et al., 2011). *MMP16* has a similar relationship to colorectal tumors (Wu et al., 2017). *MMP20*, on the other hand, is a much less investigated member of the MMP family, but was found to be expressed in colorectal tumors in a study with small number of samples (Kraus et al., 2016). This 3-way interaction (*MMP16*.rs2664369_*MMP20*.rs11225332_*MMP2*.rs11639960) had a low *p*-value (1.3E-08) in the multivariable regression analysis and is, therefore, a particularly interesting example of both the potential biological roles of MMP gene variants in disease outcomes and the potential utility multi-loci interactions to help classifying patients based on their different outcome risks.

In the analyses of the seven VEGF interaction networks (*VEGFA*, *VEGFB*, *VEGFC*, *PIGF*, *VEGFR1*, *VEGFR2*, *VEGFR3* networks), similar to MMP gene analyses, MDR programs identified generally different results (e.g. interactions and SNPs). There is not any report linking the 1 way SNP identified by both programs with colorectal or other cancers (*RFN123*.rs11130216). However, both programs were again able to identify previously unknown and significant interactions. For example, the most significant interaction associated with disease-specific survival was detected in the 3-way Cox-MDR analysis including the *CHRM3*.rs665159_*EPN1*.rs509955_*PTGER3*.rs1327460 variants (*VEGFR3* network; *p* = 2.21E-09; Table 3). All of these genes were previously linked to cancer or tumor invasion. For example, high *CHRM3* levels are linked to invasion and metastasis in colon cancers (Cheng et al., 2017; Felton et al., 2018); loss of *EPN1* was linked to elevated *VEGFR2* degradation and disorganized angiogenesis (Pasula et al., 2012); and elevated *PTGER3* levels was linked to shorter survival times in cervical cancers (Heidegger et al., 2017). On the other hand, the most significant GMDR 0.9 3-way model included variants from the *ADRB2*, *NRP1*, and *VEGFB* genes (logistic regression *p*-value = 2.4E-09; Table 4). All three genes have been shown to be associated with colorectal cancer progression (Kamiya et al., 2006; Jayasinghe et al., 2013; Ogawa et al., 2020). Also, while none of the variants identified in this study were missense or non-sense variants, according to GTEx (Lonsdale et al., 2013) and RegulomeDB (Boyle et al., 2012; RegulomeDB), a number of the SNPs identified were eQTLs (Supplementary Table S7). Together with our results in the MMP gene analysis, the fact that the identified genes and/or interacting SNPs have been previously linked to colorectal cancer and/or tumor aggression, and in some cases, are associated with gene expression levels, make these multi-loci interactions highly promising candidates for future research.

We must also comment about the MDR-based programs that we utilized in this study. Cox-MDR and GMDR 0.9, while both have proven capable of finding significant models within the datasets (albeit often different models), they vary significantly in their functionality, operation, and resource usage. Cox-MDR was provided to us by the authors as a small collection of R functions, and as such did not have the full functionality we needed for our analyses, and therefore required further efforts to run. Many of these functions/features, on the other hand, were available in GMDR 0.9, such as returning detailed outputs (including the output of high risk/low risk genotype information), the ability to set random seeds, and permutation testing. GMDR 0.9 is also readily available for download online. In contrast, an important feature possessed by Cox-MDR and missing from GMDR 0.9 is the ability to use testing balanced accuracy (TBA) score, as an alternative to CVC, to pick a best model from the cross-validation folds. GMDR 0.9 has a limitation that if two models tie for the best model among the cross-validation folds, then the model starting with the first SNP in the input dataset is chosen. This obviously has the potential to miss significant models as equally high-scoring models will be silently ignored by the software. This is an issue when using CVC to pick a best model more so than TBA (an option available in Cox-MDR), as when CVC is low it is quite likely that two or more models will tie for best model (used in GMDR 0.9; as we discuss earlier, GMDR 0.9 has missed identifying *MMP27*-rs11225388 in its 1-way analysis because of how it selects the top models (i.e. CVC and the order of data in the input files). This is rarely an issue while using TBA (that can be used in Cox-MDR) for the same purpose because as a floating point number with much higher variability than CVC, a tie is unlikely. Therefore, Cox-MDR using the TBA option overall gives results with less random model selection than GMDR 0.9, and this is an important strength of Cox-MDR. Despite its limitations, GMDR 0.9 also identified interactions that were missed by Cox-MDR.

Additionally, both Cox-MDR and GMDR 0.9 proved to have different resource usage difficulties and requirements. The Cox-MDR software cannot examine interactions in parallel, and thus, is significantly slower than GMDR 0.9. Our VEGFR2 3-way analysis of 747 SNPs took approximately 18 days to complete on the local computing cluster whereas on a similar dataset GMDR 0.9 took only 12 h. GMDR 0.9, on the other hand, has extremely large memory requirements. For the largest of our aforementioned analyses, GMDR 0.9 required a massive 220 gigabytes of RAM to complete successfully, which at the time of writing is a very large amount for a researcher to be able to obtain even on a computing cluster. In comparison, Cox-MDR only required 15 gigabytes of RAM, practically obtainable on consumer hardware. An additional resource usage issue for GMDR 0.9 is that the permutation testing procedure is performed using a Perl script external to the Java binary which contains the main program. This script uses the user’s hard drive as memory, greatly slowing down the permutation testing procedure. For a very high number of permutations this may become a significant issue. Overall, while MDR-based data reduction methods allow researchers to examine large number of interactions, in our experience, both programs have unique strengths, limitations, and feasibility concerns while examining large datasets. Therefore, while they can be considered complementary while examining SNP interactions, application of these programs widely will likely be dependent on further development.

One limitation of this study is that the patients included are all of Caucasian ancestry. We also limited our work to common SNPs and genes from autosomal chromosomes, therefore, the potential interactions among rare SNPs and MMP/VEGF-interactor genes located in X or Y chromosomes remain unexamined. Our results are exploratory, therefore replication studies are needed to confirm whether these SNPs/interactions have prognostic value in the clinic. The genes were limited to select genes related to cancer and progression, therefore further studies are needed to examine the potential interactions in other genes/interaction networks. Our study also has several strengths. This is one of the first studies that applied MDR-based approaches while examining survival outcomes in colorectal cancer, and the first one, in our knowledge, that examined such relatively large number of interactions (∼90 million). We explored and applied two different MDR-based programs, one using the survival times (Cox-MDR) and the other 5-years survival status (GMDR 0.9) with a slightly different methodology that allowed us to comprehensively examine the interactions and compare the programs’ utility. The patient cohort is a well annotated cohort. Additionally, the use of cross-validation and permutation testing, as well as the repeating the Cox-MDR/GMDR 0.9 runs (20 times) to identify the most consistent best models (called top models in this study) were critical and helped reduce the false-positive findings. More importantly, our results demonstrated that MDR can be powerful in detecting interactions among genetic variants in prognostic studies and the novel 2-way and 3-way SNP interactions identified in this study bring a new depth to colorectal cancer and prognostic research.

In conclusion, we performed a two-part study applying two MDR-based programs to examine the SNP interactions in relation to patient outcomes in colorectal cancer. Our work indicates that MDR-based programs can be quite useful in examining the interactions among the genotypes/SNPs while examining the novel prognostic markers in colorectal cancer. Our results also suggest the presence of novel SNPs and interactions in MMP and VEGF family genes that are associated with the patient outcomes in colorectal cancer. These SNPs are excellent candidates for further biomarker studies.

## Data Availability

The datasets presented in this article are not readily available. Data that support the findings of this study are available from the Newfoundland Colorectal Cancer Registry/Memorial University. However, restrictions apply to the availability of this data, and so data are not publicly available. The data used in this study cannot be made publicly available as patients were not consented to make their data publicly available or accessible. Clinical and genetic data are available from the Newfoundland Colorectal Cancer Registry (NFCCR) upon reasonable request for researchers who meet the criteria for access to confidential data. Request to access the datasets should be directed to Newfoundland Colorectal Cancer Registry (PP; pparfrey@mun.ca) and Research, Grant, and Contract Services (rgcs@mun.ca) at Memorial University of Newfoundland, St. John’s, NL, Canada, and the ethics approval shall be obtained from the Health Research Ethics Board (HREB), Ethics Office, Health Research Ethics Authority, Suite 200, 95 Bonaventure Avenue, St. John’s, NL, A1B 2X5, Canada. The Cox-MDR code can be requested from Dr. Seungyeoun Lee. The GMDR 0.9 code can be requested from the developers, Drs. Xiang-Yang Lou, Jun Zhu, or Ming D. Li.
