# Supplementary material for: Examining SNP-SNP interactions and risk of clinical outcomes in colorectal cancer using multifactor dimensionality reduction based methods
Source: Front Genet. 2022 Aug 3;13:902217. doi: 10.3389/fgene.2022.902217 (PMC9385108; doi:10.3389/fgene.2022.902217)
Supplement: Supplementary file 1 [file DataSheet1.PDF]

Supplementary Material

1 Methods

Part 1. Analysis of MMP gene SNPs

1.1 Patient cohort

Table S1: Baseline characteristics of the 439 patients who are included in the Part 1 of the study.

**MMP Project**		
**Variable**	**N**	**%**
**Age at Diagnosis**	Median: 62; Range: 21 – 75 years	
**Disease Stage**		
I	72	16.40
II	174	39.64
III	146	33.26
IV	47	10.71
**MSI Status**		
Stable/MSI-low	389	88.61
MSI-high	50	11.39
**Tumor Location**		
Colon	298	67.88
Rectum	141	32.12
**#OS Time**	Median: 7; Range: 0 – 11 years	
**#OS Status**		
Alive	279	63.55
Dead	160	36.45
**##5-Year OS Status**		
Alive at 5 years	324	73.80
Dead at 5 years	115	26.20

MSI: Microsatellite instability; **OS:** Overall survival. #Used in Cox-MDR, Kaplan-Meier, and Cox regression analyses. ##Used in GMDR 0.9 and logistic regression analyses

1.2 Genes Selected for Part 1

Genomic locations for MMP genes (n=23) were identified using the UCSC genome browser (Kent et al. 2002). When needed, alternate gene symbols were found using the HUGO gene nomenclature (HGNC) database (Yates et al., 2017). Of the results returned upon searching for each MMP gene in the UCSC genome browser, the earliest start location among the UCSC gene locations listed was selected as the beginning of the genomic range and the latest end location among locations listed was chosen as the end of the genomic range (**Table S2**).

Table S2: MMP genes, their genomic locations, and the numbers of SNPs per gene that are included in Part 1 of this study

Gene	Chromosome	Genomic Range (bp)	Number of SNPs	SNPs
MMP1	11	102660641 - 102668966	7	rs2239008_A rs470558_A rs10488_A rs470215_G rs1938901_T rs7125062_C rs3213460_A
MMP2	16	555113081 - 55540586	16	rs2287074_A rs11639960_G rs1477017_G rs865094_G rs17301608_T rs1132896_C rs1053605_T rs866770_G rs9302671_T rs243845_T rs243843_G rs243842_C rs183112_A rs2287076_C rs243835_T rs10775332_T
MMP3	11	102706528 - 102714342	3	rs3025066_G rs3020919_T rs679620_A
MMP7	11	102391239 - 102401478	4	rs17886371_G rs14983_T rs2156528_A rs1996352_C
MMP8	11	102582526 - 102595685	8	kgp5394892_G rs1940475_C rs12365082_A rs7934972_A rs3740938_A rs2012390_C rs12803000_G rs2155052_C
MMP9	20	44637547 - 44645200	4	rs2274755_T rs17576_G rs2274756_A rs20544_C
MMP10	11	102641233 - 102651359	8	rs470168_A rs12290253_C rs547561_G rs12272341_A rs4431992_C rs2276108_G rs17860950_C rs486055_T

MMP11	22	24115036 - 24126503	3	rs738791_T rs2267029_A rs738792_C
MMP12	11	102733464 - 102745764	2	rs17368582_C rs11225442_A
MMP13	11	102813721 - 102826463	3	rs10502009_G rs3819089_A rs640198_A
MMP14	14	23305793 - 23316803	8	rs1042703_C rs762052_A rs8006914_T rs2236302_G rs1042704_A rs2236307_C rs743257_T rs17882342_D
MMP15	16	58059282 - 58080804	0	
MMP16	8	89049460 - 89339717	56	rs10504847_T rs2664369_G rs2664370_C rs17719609_C rs16877270_G rs1477908_C rs10103111_T rs2616493_C rs10098052_A rs2664346_C rs2616488_C rs6469206_G rs7826929_G rs2616506_C rs17663841_C rs977231_G rs2664352_C rs11782395_A rs1477916_T rs17664125_C rs13277637_T rs4961076_C rs9297422_C rs1382105_T rs1477917_G rs2664361_C rs16878818_T rs10099888_C rs7819728_A rs1996637_C rs1519938_G rs6981717_C rs2176771_C rs1519942_G rs12546847_C rs4961080_C rs13261974_A rs6469298_T rs17666351_G rs13261169_T rs1401861_A rs1879201_G rs17666490_T rs16880099_A rs7826477_T rs6994019_T rs16880416_T rs2222294_T rs7817382_G rs7834743_A rs7816934_C rs7000030_T rs3851539_G rs10504846_A rs10094702_C rs7835845_T
MMP17	12	132312941 - 132336316	11	rs3087864_G rs4964924_T rs4964927_T rs11246838_G rs6598163_A rs34515698_T rs7300198_C rs12099648_A rs9634312_A rs11613757_T rs11835665_A
MMP19	12	56229214 - 56236767	3	rs2242295_A rs2291267_A rs2291268_G

MMP20	11	102447566 - 102496063	17	rs2292730_A rs11225332_C rs1711399_C rs1711433_G rs10895322_G rs1711430_T rs1711427_C rs1784425_G rs1784424_A rs3781787_C rs3781788_T rs17098913_A rs10502005_A rs2280211_C rs11225344_A rs1962082_T rs2245803_A
MMP21	10	127455027 - 127464390	3	rs7922546_A rs10901424_T rs12775804_A
MMP23B	1	1567560 - 1570030	0	
MMP24	20	33814539 - 33864804	21	kgp4728036_A kgp4471741_A kgp6966600_G kgp481229_T kgp2046320_G kgp7289875_G kgp5576338_T kgp10149373_G kgp7633769_A kgp9807173_C kgp1472099_T rs12479765_A rs2425022_C rs6088776_C rs2247828_G rs2425024_C rs2254207_C rs11696548_T rs6060341_G rs7280_G rs2425032_C
MMP25	16	3096682 - 3110724	7	rs2247226_T rs10431961_T rs7199221_A rs1064875_T rs1064948_A rs11864930_A rs10438593_T
MMP26	11	5009424 - 5013659	1	rs2499958_A
MMP27	11	102562415 - 102576468	15	rs12099177_A rs2509010_T rs11607205_A rs1276289_A rs1276286_T rs2846723_C rs2846701_G rs2846703_G rs3809018_A rs17099425_G rs11225386_G rs11225388_G rs2846707_A rs1939015_G rs11225389_A
MMP28	17	34083269 – 34122640	1	rs3826404_G

bp: base pair; **SNP**: Single Nucleotide Polymorphism.

1.3 Data Considerations

We have taken a number of measures while preparing the data files for analysis. For example, Cox-MDR automatically rounds numbers to the nearest integer; hence, we rounded the continuous variables (age, overall survival (OS) time). Note that the rounded OS time was used to determine the 5-year survival status that was used in the GMDR 0.9 analysis. Additionally, at least Cox-MDR requires a complete dataset (i.e. no missing data). To address this data requirement, we only included the patients with complete clinical data (n=439) and SNPs/polymorphisms with zero missing genotype rate.

1.4 Code Extension and Runs

We added to the Cox-MDR code the ability to retrieve genotype and training balanced accuracy values for each Cox-MDR model, perform multiple runs at once, utilize random seed setting/loading (e.g. required for permutation testing purposes), and conduct permutation testing for any selected model. After these extensions, Cox-MDR code was tested to examine its features and to ensure that it worked correctly and produced the correct output.

As part of this study, the permutation testing Perl script included with GMDR 0.9 was extended. Specifically, lines 160 and 213 were edited to allow the setting of random seeds, adding the parameter "-seed=<long>" as specified by the GMDR-0.9 --help command-line argument output.

Cox-MDR and GMDR 0.9 programs were run in R(R Core Team, 2017; Microsoft R Open, 2019) (R versions 3.5.0, 3.5.1, 3.6.2; Microsoft R Open version 3.5.1) and Java respectively. Cox-MDR and GMDR 0.9 analyses on large interaction datasets were performed in parallel to reduce computational time using the hardware and software systems at the Center for Health Informatics and Analytics (CHIA), Memorial University of Newfoundland. For Cox-MDR analyses in CHIA, we used manually set seeds (generated in R) in order to ensure parallel runs had different cross-validation partitioning despite starting at roughly the same time, as by default the random number generation of R sets a random seed based on system time.

1.5 Permutation Testing

For Cox-MDR permutation testing, an R function was written and run through R-Studio (RStudio Team, 2015). This function randomly shuffles specified columns of the input data and can be called multiple times to produce different shuffles of the dataset. This function was designed so that, similar to GMDR 0.9's permutation testing procedure**,** elements in shuffled columns still remained together in the same row, but the relationship between these elements and all remaining elements (i.e. SNP genotypes) was randomized. For the permutation testing procedure, the Cox-MDR or GMDR 0.9 method was applied to 1000 random shuffles of the data. The permutation testing procedure ran the Cox-MDR or GMDR 0.9 program using the same random seed as the run (i.e. the run that identified the top MDR model) being tested to ensure the same patients were in the same cross-validation folds between runs. After 1000 runs, the p-value was determined to be the number of testing balanced accuracy (TBA) values for Cox-MDR, or average (among cross-validation folds) TBA values for GMDR 0.9, which were as high as or higher than the observed TBA value for the "top model" divided by the number of permutations (n=1000); if this value was <= 5%, then the top MDR model was deemed to be significant (Ritchie et al., 2001) (i.e. not likely to be detected by chance). The permutation testing procedure for GMDR 0.9 functions identically to that of Cox-MDR, except that

GMDR 0.9 software uses the average TBA value among the 5 cross validation folds instead of the highest TBA value.

For larger datasets using GMDR 0.9 performing the permutation testing procedure on a desktop computer exceeded hardware resources. For these sets, permutation testing was performed on the CHIA computing cluster.

Part 2: ***Interactions among the SNPs of VEGF interaction network genes***

Table S3: Baseline characteristics for the 400 patients included in the VEGF interactome study

**VEGF Project**		
**Variable**	**N**	**%**
Age At Diagnosis	Median: 61; Range 21- 75 years	
**Disease Stage**		
I	77	19.25
II	165	41.25
III	126	31.50
IV	32	8.00
**MSI Status**		
Stable/MSI-low	350	87.5
MSI-high	50	12.5
**Tumor Location**		
Colon	264	66.00
Rectum	136	34.00
**Baseline Radiation**		
Adjuvant	100	25.00
Others	300	75.00
**Baseline Chemotherapy**		
Adjuvant	223	55.75
Others	177	44.25
**DSS Time**	Median: 14; Range: 0 -19 years	
**#DSS Status**		

Alive	309	77.25
Dead	91	22.75
**##5-Year DSS Status**		
Alive at 5 years	337	84.25
Dead at 5 years	63	15.75

MSI: Microsatellite instability; **DSS**: Disease Specific Survival. #Used in Cox-MDR, Kaplan-Meier and Cox regression analyses. ##Used in GMDR 0.9 and logistic regression analyses. Note that this table includes the 5 patients, who were removed from GMDR 0.9 analysis

1.6 Identification of Interaction Partners of the VEGF Family Genes

For the BioGRID (Stark et al., 2006; Oughtred et al., 2021; BioGRID | Database of protein, chemical, and genetic interactions) searches, species was set to "Homo sapiens". Interactions were downloaded in BioGRID TAB 2.0 format. Note that BioGRID uses these aliases for the following VEGF family proteins in their records: FLT1 for VEGFR1, KDR for VEGFR2, FLT4 for VEGFR3, and PGF for PIGF. Chemical interactions were filtered out of results of BioGRID searches before producing BioGRID TAB files and network diagrams (**Figure S1**). Non-human interactors were removed from the interactor datasets. A set of interactors for each VEGF family gene was produced by combining the columns "Official Symbol Interactor A" and "Official Symbol Interactor B" for the BIOGRID TAB files and removing duplicate gene symbols. The number of interactors of each VEGF gene are given in **Table S4A**.

In cases where multiple gene symbols had the same genomic location, we kept only the symbol which was found in our BioGRID search.

While using legacy Biomart section to obtain genomic locations for interactors, genes that were located on the X chromosome were excluded from further analysis (**Table S4A**). Additionally, entries with "PATCH" annotations were removed from analysis, as were entries with unusual annotations, opting for locations from the UCSC under the "Comprehensive Gene Annotation Set from GENCODE Version 19" heading. It was confirmed that the same gene in different interaction sets had the same genomic location.

Figure S1: Interaction networks for VEGFA, VEGFB, VEGFC, VEGFR1, VEGFR2, VEGFR3, and PIGF taken from Biogrid.

Figure S1 A: VEGFA interaction network.

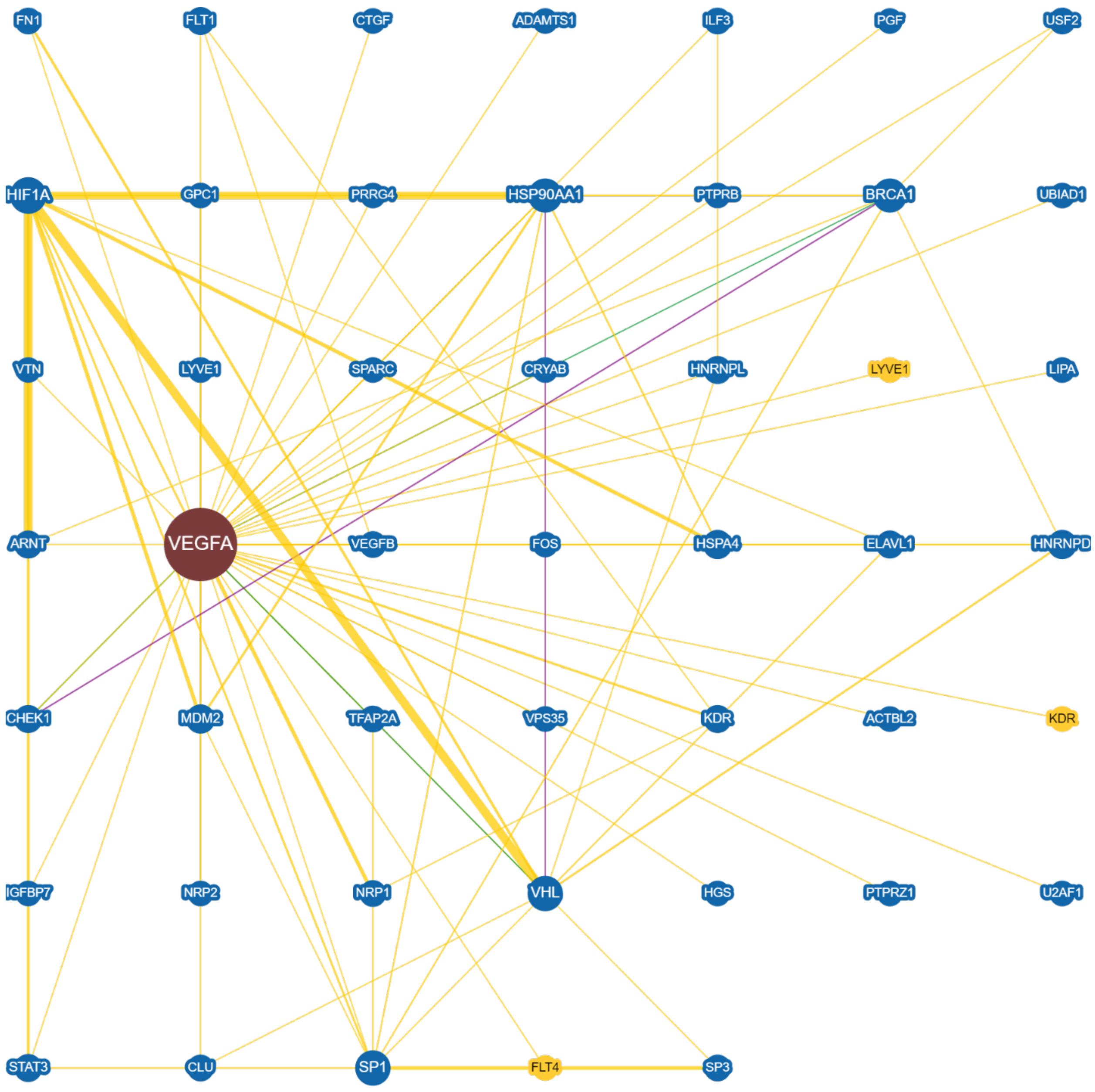

Figure S1 B: VEGFB interaction network.

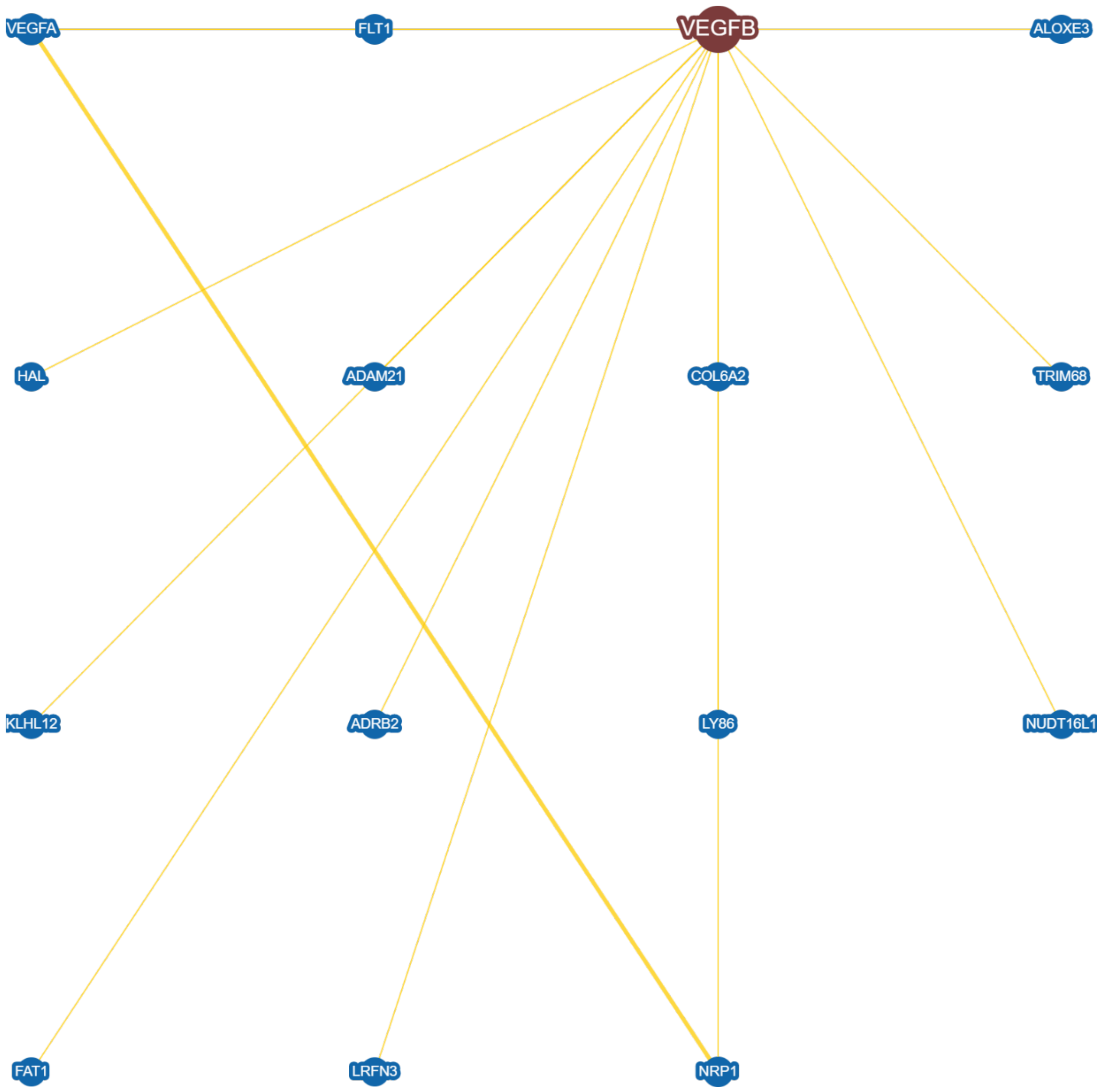

Figure S1 C: VEGFC interaction network.

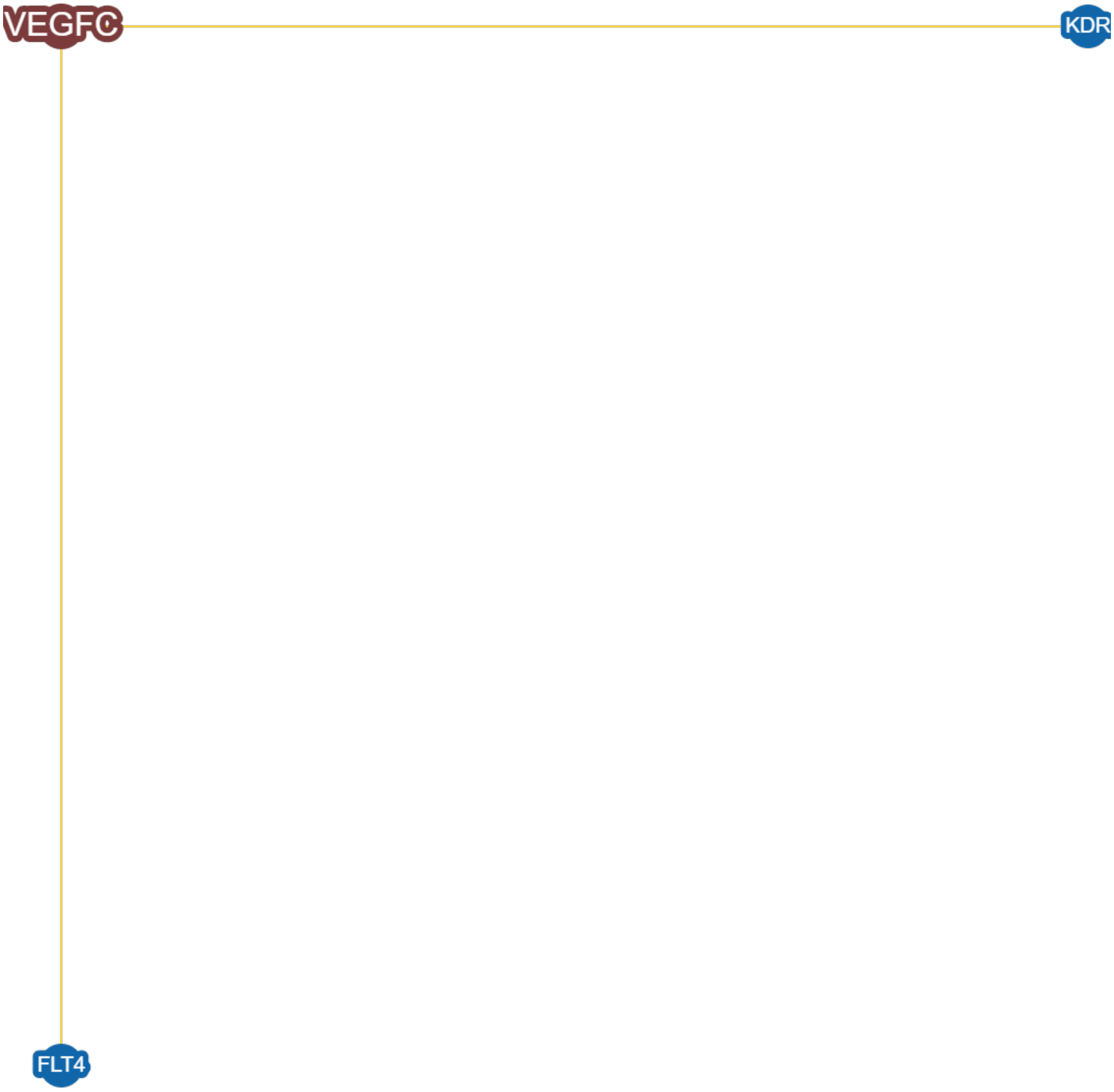

Figure S1 D: VEGFR1/FLT1 interaction network.

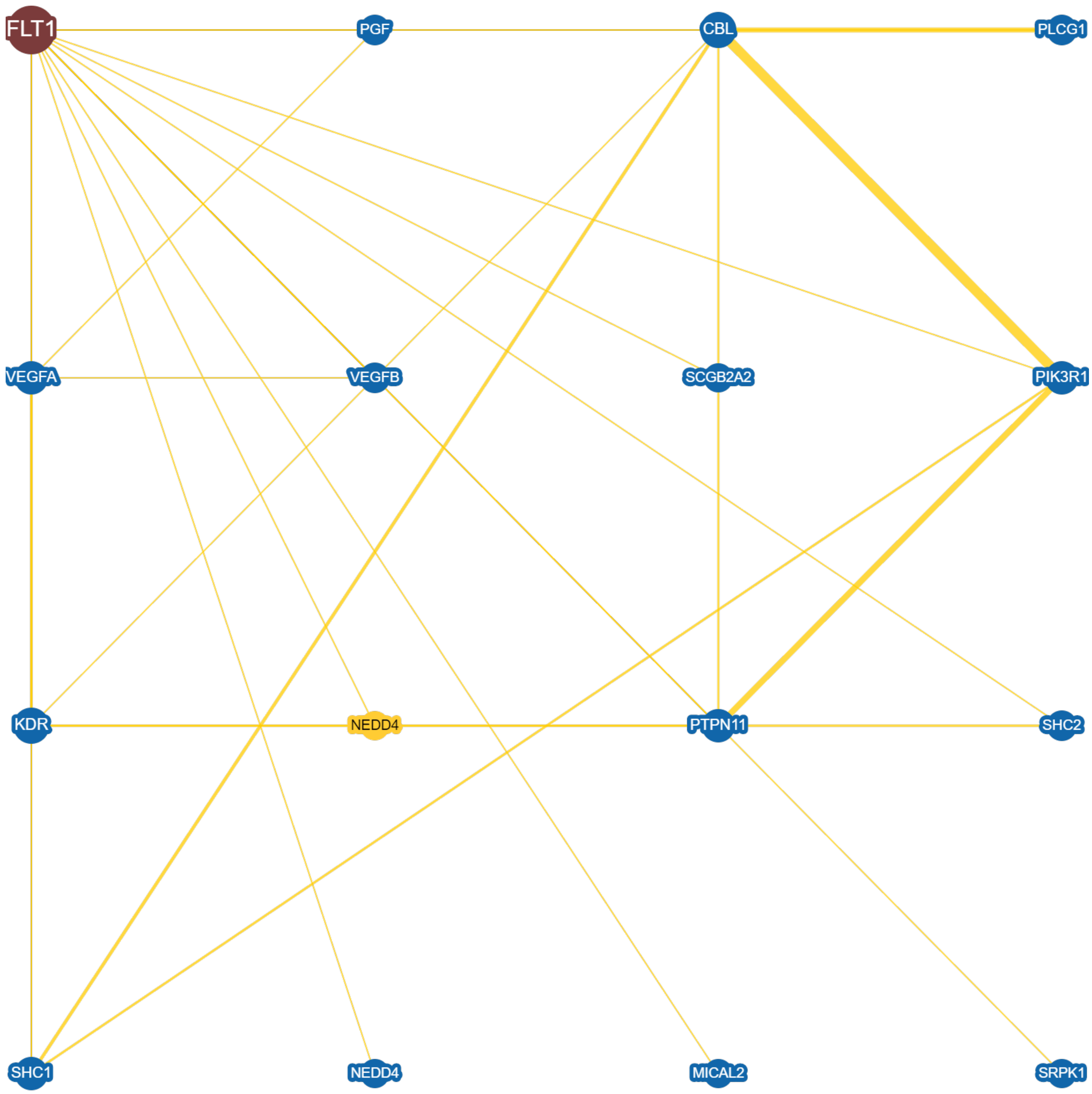

Figure S1 E: VEGFR2/KDR interaction network.

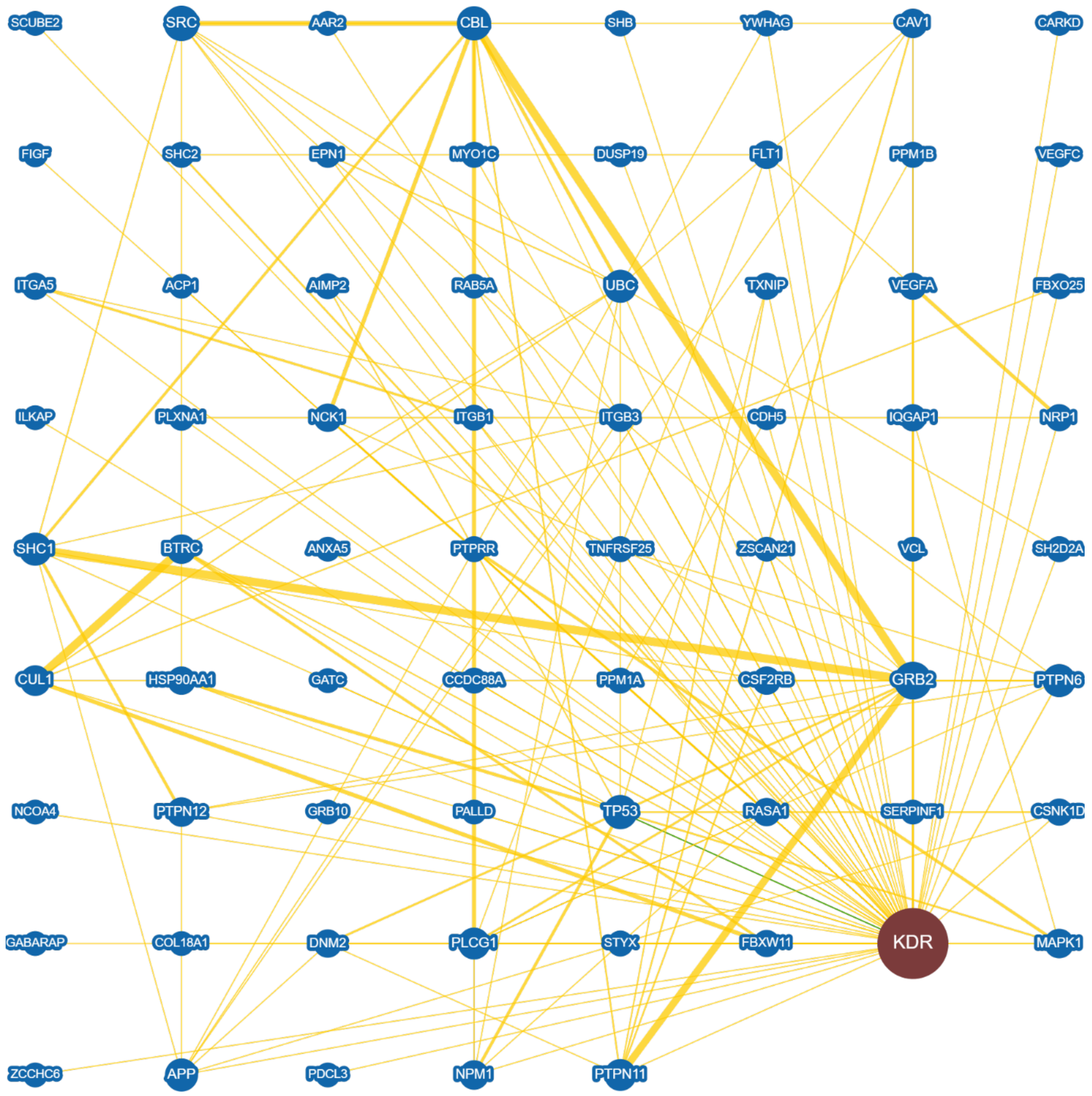

Figure S1 F: VEGFR3/FLT4 interaction network.

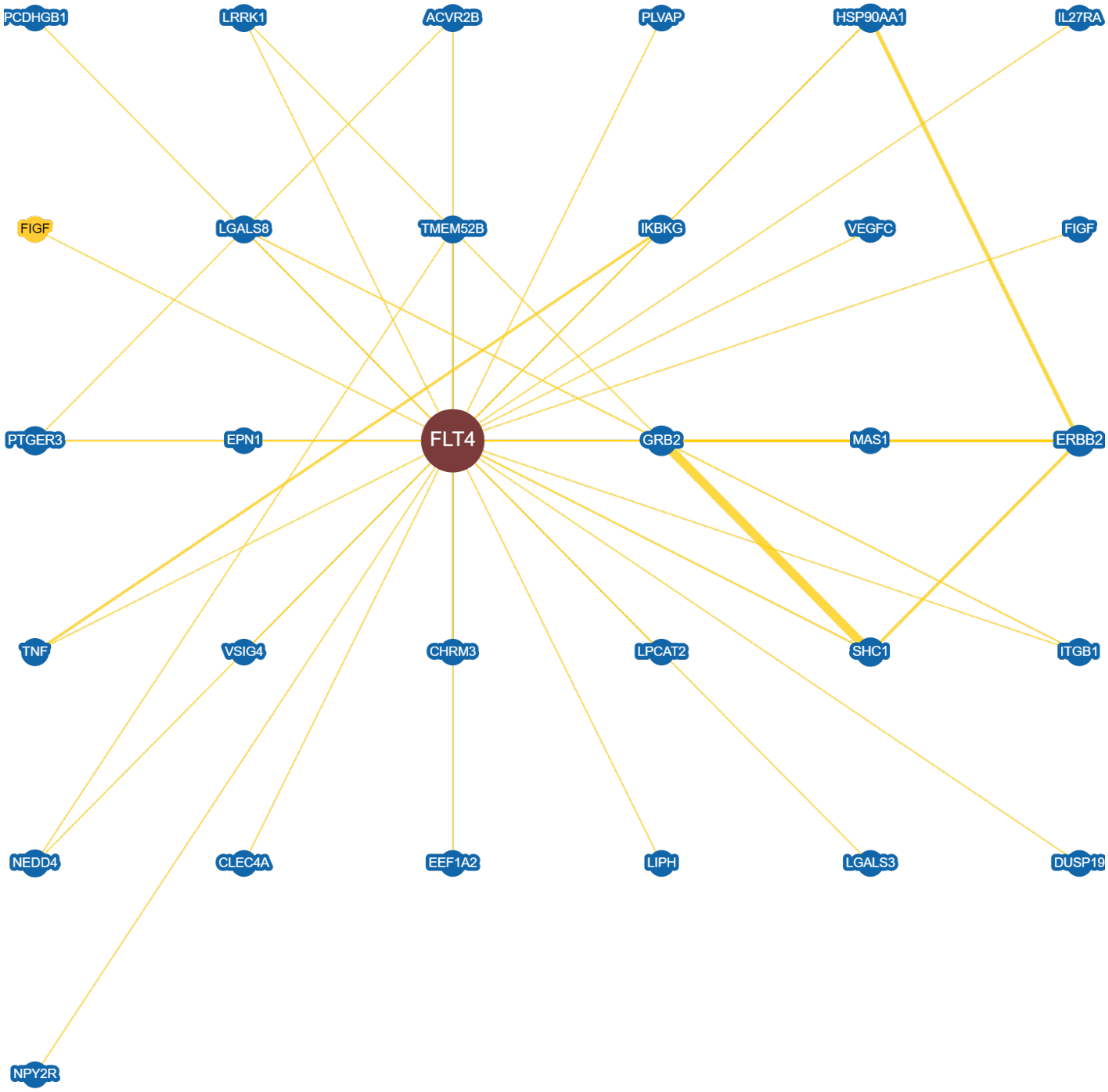

Figure S1 G: PIGF/PGF interaction network.

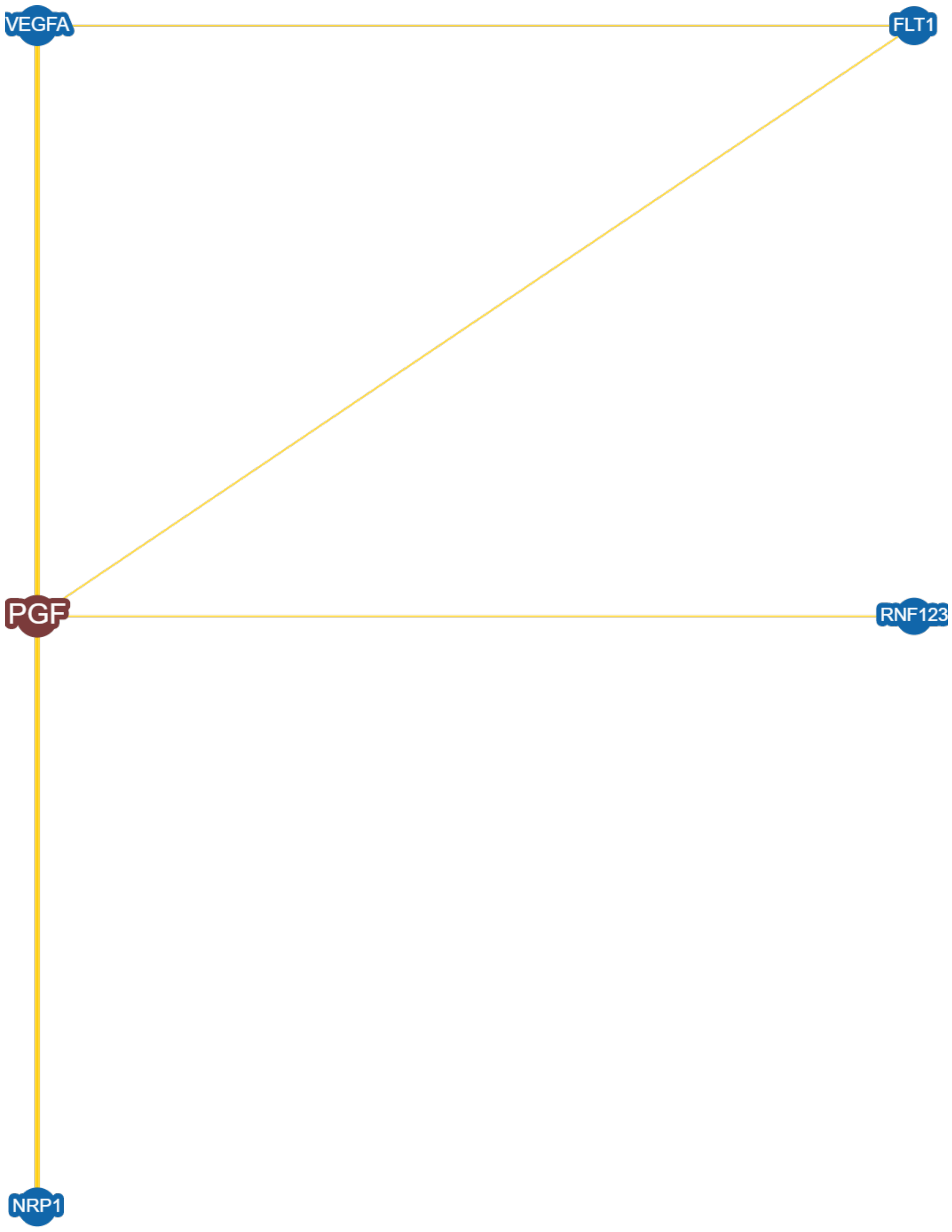

Network diagrams generated using BioGRID (Oughtred et al., 2021; BioGRID | Database of protein, chemical, and genetic interactions). Line size represents the number of unique interactions in the BioGRID database for a given pair of interactors. Yellow lines represent physical interactions, green lines represent genetic interactions, and purple lines represent evidence of both physical and genetic interactions. Yellow nodes represent non-human genes which were subsequently removed from analysis. Aliases: FLT1, KDR, FLT4, and PGF refer to VEGFR1, VEGFR3, VEGFR3, and PIGF respectively.

Table S4 A: Number of VEGF interactors in each network.

VEGF family member protein	Number of genes in the interaction network	Number of genes in the interaction network*
VEGFA	43	43
VEGFB	14	14
VEGFC	3	3
PIGF	5	5
VEGFR1	15	15
VEGFR2	69	68
VEGFR3	26	23
**TOTAL**	**175**	**171**

*After the genes on the X chromosome are excluded.

Table S4 B: Final list of genes included in study of VEGF family interactome networks

VEGF family member protein	Final list of genes included in study
VEGFA	ACTBL2 ADAMTS1 ARNT BRCA1 CHEK1 CLU CRYAB ELAVL1 FLT1(VEGFR1) FN1 FOS GPC1 HGS HIF1A HNRNPD HNRNPL HSP90AA1 HSPA4 IGFBP7 ILF3 KDR(VEGFR2) LYVE1 MDM2 NRP1 NRP2 PGF(PIGF) PRRG4 PTPRB PTPRZ1 SP1 SP3 SPARC STAT3 TFAP2A U2AF1 USF2 VEGFA VEGFB VHL VPS35 VTN
VEGFB	ADAM21 ADRB2 ALOXE3 COL6A2 FAT1 FLT1 HAL KLHL12 NRP1 TRIM68 VEGFA VEGFB
VEGFC	FLT4(VEGFR3) KDR(VEGFR2) VEGFC
VEGFR1	CBL FLT1(VEGFR1) KDR(VEGFR2) MICAL2 NEDD4 PGF(PIGF) PIK3R1 PLCG1 PTPN11 SCGB2A2 SHC2 SRPK1 VEGFA VEGFB
VEGFR2	AAR2 ACP1 AIMP2 ANXA5 APP BTRC CARKD CAV1 CBL CCDC88A CDH5 COL18A1 CSF2RB CSNK1D CUL1 DNM2 DUSP19 EPN1 FBXO25 FBXW11 FLT1(VEGFR1) GABARAP GATC GRB2 GRB10 HSP90AA1 ILKAP IQGAP1 ITGA5 ITGB1 ITGB3 KDR(VEGFR2) MAPK1 MYO1C NCK1 NCOA4 NPM1 NRP1 PALLD PDCL3 PLCG1 PLXNA1 PPM1A PPM1B PTPN6 PTPN11 PTPN12 PTPRR RAB5A RASA1 SCUBE2 SERPINF1 SH2D2A SHB SHC2 SRC STYX TNFRSF25 TP53 UBC VCL VEGFA VEGFC YWHAG ZCCHC6 ZSCAN21
VEGFR3	CHRM3 DUSP19 EEF1A2 EPN1 ERBB2 FLT4(VEGFR2) GRB2 HSP90AA1 ITGB1 LGALS3 LGALS8 LIPH LRRK1 MAS1 NEDD4 NPY2R PCDHGB1 PLVAP PTGER3 TMEM52B TNF VEGFC

PIGF	FLT1 NRP1 PGF(PIGF) RNF123 VEGFA

Parentheses indicate alternate gene symbols used in this document

Table S5: All genes and SNPs included in VEGF interactor study

Gene	Number of SNPs	SNPs
AAR2	3	rs2425193 rs2104007 rs2425202
ACP1	1	rs7584915
ACTBL2	1	rs13159014
ADAM21	3	rs11622815 rs2000352 rs4143920
ADAMTS1	4	rs9636786 rs13615 kgp10200667 rs370850
ADRB2	3	rs1042711 rs1042713 rs1042717
AIMP2	6	rs1860461 rs1860460 rs6979676 rs7803611 rs7781199 rs4560
ALOXE3	10	rs3809882 rs4792214 rs6503080 rs9894356 rs2289587 rs7215658 rs3027209 rs4414548 rs4792239 rs3027229
ANXA5	7	rs11098637 rs12511956 rs10518391 rs6534309 rs6857766 rs13145977 rs2306416
APP	45	rs214486 rs3787620 rs2829973 rs1876064 rs454017 rs1787438 rs17001492 rs1783016 rs214488 rs2829984 rs2234983 rs216779 rs367489 rs440666 rs2014146 rs216762 rs1701000 rs9305268 rs128647 rs2096488 rs8132200 rs12626960 rs7278838 rs2830008 rs7281216 rs768040 rs2070655 rs2830028 rs2830034 rs2830038 rs1041420 rs7283136 rs2830044 rs2070654 rs2830051 rs2830052 rs11702267 rs2830067 rs2830071 rs2830088 rs17588612 rs455465 rs458848 rs4817090 rs2830101
ARNT	5	rs10847 rs10305710 rs2228099 rs2134688 rs11204737
BRCA1	5	rs8176305 rs3737559 rs1799950 rs799923 rs799912
BTRC	9	rs7090670 rs10786634 rs7901883 rs4451650 rs9419913 rs9420839 rs17767748 rs4151060 rs11595968
CARKD	2	rs330550 rs179356

CAV1	10	rs926198 rs10256914 rs3807986 rs959173 rs3807989 rs3815412 rs1022436 rs9920 rs1049334 rs1049337
CBL	5	rs6589722 rs1893032 rs2511844 rs11217234 rs1052121
CCDC88A	9	rs2576692 rs1047948 rs1545121 rs4484068 rs3099084 rs10496042 rs11684805 rs6721972 rs6545492
CDH5	11	rs10852432 rs1077318 rs1076019 rs2344564 rs7499886 rs2344565 rs1130844 rs11640843 rs1073584 rs16956504 rs1972839
CHEK1	6	rs3731395 rs10893405 rs521102 rs2282535 rs11220181 rs11220182
CHRM3	94	rs4130463 rs10925877 rs12123857 rs6678395 rs12137225 rs10802767 rs6676664 rs6687984 rs17645304 rs12086449 rs10925888 rs16838380 rs1782349 rs1782357 rs12041334 rs10925907 rs16838444 rs17646815 rs7525710 rs11804608 rs6670728 rs12135445 rs13376565 rs6429140 rs12119540 rs6429144 rs12120382 rs17594385 rs6685121 rs6688669 rs6690612 rs2355230 rs726168 rs12088787 rs12037424 rs6691263 rs10925941 rs12090480 rs10802789 rs1867266 rs1867265 rs6692711 rs12406493 rs4145784 rs2278642 rs1431718 rs12124903 rs10925971 rs685475 rs685550 rs685960 rs843030 rs6703930 rs7533134 rs17657156 rs2841037 rs481036 rs4266870 rs483411 rs693948 rs665159 rs1111249 rs12059546 rs558438 rs6690809 rs7543259 rs6429157 rs1578180 rs1934349 rs7536133 rs6698105 rs589962 rs685548 rs602117 rs1125489 rs1594513 rs10925994 rs682355 rs536477 rs2217533 rs10802812 rs16839034 rs16839045 rs10926008 rs16839051 rs10926009 rs7527677 rs10399860 rs12036109 rs7520974 rs6701181 rs6682184 rs4431831 rs4659554
CLU	4	rs9331947 rs7812347 rs7982 rs9331888
COL18A1	31	rs879330 rs8128168 rs2026886 rs4819099 rs4819101 rs2838916 rs2838917 rs2838920 rs2838923 rs8126757 rs11702782 rs7275991 rs9980531 rs4819115 rs2236451 rs11702494 rs2230688 rs2236459 rs2838942 rs2246749 rs11702425 kgp9623698 kgp383228 rs2236475 rs7279445 rs3753019 rs2236483 rs12483553 rs7278425 rs17004785 rs7867
COL6A2	10	rs9978018 rs2839108 rs17357592 rs2839112 rs2839113 rs7280485 rs2839116 rs3088026 rs1044598 rs2839117

CRYAB	3	rs4252588 rs11214040 rs11214043
CSF2RB	9	rs2075726 rs5756407 rs6000488 rs11089810 rs909486 rs1534882 rs11705394 rs131840 rs131842
CSNK1D	2	rs11653735 rs4789846
CUL1	13	rs243551 rs243538 rs243524 rs243523 rs11760399 rs243492 rs243482 rs243477 rs1014095 rs3823635 rs10271133 rs7779159 rs2007404
DNM2	10	rs12974306 rs4334414 rs714307 rs892086 rs12232826 rs4804524 rs7246673 rs2278444 rs2287029 rs12461992
DUSP19	7	rs16823976 rs3748880 rs12463411 rs11883456 rs2705730 rs17704934 rs2944346
EEF1A2	5	rs2274860 rs2750395 rs310619 rs8126435 rs910948
ELAVL1	8	rs2042920 rs12983784 rs4804244 rs759817 rs10401186 rs3786619 rs7251814 rs1204494
EPN1	7	rs8104242 rs3786642 rs10408454 rs10410404 rs6509955 rs7255531 rs2287831
ERBB2	6	kgp11187652 kgp8452497 kgp8195839 rs4252612 rs1801200 rs4252667
FAT1	31	rs3775309 rs1280092 rs2637777 rs1280103 rs28647489 rs2249916 rs2249917 rs3733406 rs11931107 rs189031 rs7663350 rs328432 rs328431 rs1388297 rs7672047 rs162062 rs167853 rs2130910 rs10155467 rs2130909 rs2375889 rs162182 rs4862723 rs327080 rs455600 rs907986 rs455219 rs13123522 rs3733414 rs1491248 rs327102
FBXO25	15	rs13279681 rs17812876 rs17665364 rs17064974 rs13340594 rs1530662 rs3735925 rs10092971 rs2278765 rs13253643 rs10088894 rs12546599 rs10503146 rs10109251 rs17665621
FBXW11	3	rs9313563 rs9313564 rs9313565

FLT1	36	rs9554314 rs12429309 rs9513070 rs12877323 rs3794397 rs3794399 rs2296188 rs2296189 rs7987291 rs7987649 rs3794400 rs2387632 rs3936415 rs17086609 rs1853581 rs7989623 rs7995976 rs9551462 rs3751395 rs17086617 rs17537350 rs7332329 rs9508021 rs9513099 rs11149523 rs9508034 rs9513112 rs9554330 rs3794405 rs9513113 rs10507386 rs585421 rs622227 rs655024 rs679791 rs598945
FLT4	16	rs307822 rs2279622 rs11739750 rs2242217 rs400330 rs3797104 rs307823 rs3797102 rs3736061 kgp53910 rs2290983 rs10085025 rs4700745 rs10072977 rs11748431 rs307814
FN1	18	rs1263 rs11651 kgp9543736 rs2289200 rs6707530 rs7608342 rs13652 rs1250201 rs7588661 rs11883812 rs1561302 rs7596677 rs17516906 rs724617 rs1437799 rs16854041 rs7609476 rs1250246
FOS	2	rs7101 rs1063169
GABARAP	2	rs11656323 rs222843
GATC	4	rs17431446 rs2235217 rs7957424 rs3847971
GPC1	13	rs7577243 rs13424854 rs7589322 rs3828334 rs3828336 rs2292832 rs881029 rs12695020 rs2228327 rs1126920 rs13013933 rs3792215 rs1042823
GRB10	32	rs4245556 rs4947406 rs4947709 rs2715129 rs11770199 rs17544225 rs2250152 rs2299150 rs980716 rs6948959 rs2715117 rs17544971 rs2237444 rs6593077 rs2237447 rs17133917 rs2237456 rs1800504 rs2237477 rs2237482 rs10248619 rs2282930 rs2299155 rs17152102 rs2108349 rs6968827 rs1024532 rs6979369 rs7805310 rs6976572 rs7791286 rs6593185
GRB2	3	rs16967789 rs959260 rs4789182
HAL	14	rs1059845 rs2230885 rs11108358 rs7297245 rs10492228 rs3213737 rs12319274 rs12307364 rs10745747 rs17676826 rs10859997 rs10492227 rs2302629 rs2302628
HGS	3	kgp785391 rs6565620 rs34384005
HIF1A	5	rs2301106 rs10129270 rs4899056 rs12434438 rs2057482

HNRNPD	3	rs2288338 rs1820577 rs1365872
HNRNPL	3	rs10403012 rs2278012 rs862456
HSP90AA1	5	rs7155973 rs3736807 rs11621560 rs10873531 rs1190603
HSPA4	3	rs13161158 rs11749966 rs14355
IGFBP7	35	rs1277308 rs11573128 rs2271808 rs1277311 rs11133472 rs1718885 rs7687211 rs6852762 rs3821996 rs6554404 rs881382 rs1713973 rs1401189 rs1713963 rs11573086 rs1713959 rs7656865 rs1277293 rs7356193 rs17761305 rs1718856 rs4865174 rs10516163 rs11934877 rs1714014 rs1718848 rs1714011 rs1718845 rs11936912 rs10019698 rs1718858 rs1718861 rs6851308 rs4865181 rs10004910
ILF3	2	rs2569507 rs13465
ILKAP	6	rs2278737 rs2880132 rs2880131 rs6431588 rs2305171 rs3795903
IQGAP1	7	rs17176602 rs6496674 rs12912995 rs16974212 rs11853271 rs9944285 rs3539
ITGA5	3	rs7306692 rs1270919 kgp6380544
ITGB1	16	rs2153875 rs2488320 rs2230396 rs3780873 rs10763902 rs10827163 rs10827164 rs1009002 rs11009157 rs1187078 rs2457705 rs1187095 rs2475193 rs10827167 rs1187086 rs11591508
ITGB3	12	rs10514919 rs7209700 rs11868894 rs2292867 rs8073229 rs5918 rs2292699 rs12603582 rs3809863 rs7225700 rs12948299 rs11867160
KDR	14	rs12642307 rs2125489 rs1531289 rs17709898 rs6838752 rs6828477 rs11732292 rs17085326 rs2034965 rs17711073 rs2305948 rs7692791 rs6837735 rs12502008
KLHL12	3	rs12089566 rs4950887 rs2275734
LGALS3	2	rs7160523 kgp43854

LGALS8	15	rs17753447 rs1266381 rs10802546 rs4659682 rs10925157 rs1266384 rs12041958 rs2799426 rs10925158 rs3754245 rs2472126 rs11807205 kgp6759139 rs2298096 rs2298098
LIPH	3	rs6788865 rs9790230 rs4626118
LRRK1	38	rs12148466 rs11630691 rs11858394 rs4075387 rs7170683 rs12441903 rs4965738 rs4965741 rs721906 rs8038607 rs12915954 rs7176253 rs2412000 rs12914811 rs12439038 rs966293 rs11857262 rs6598411 rs1993375 rs12595297 rs878274 rs6598412 rs1078513 rs2034809 rs963333 rs930847 rs11633278 rs11247253 rs4427776 rs12594881 rs12592409 rs4965778 rs4965780 rs11857803 rs17161155 rs17744500 rs2925202 rs1048327
LYVE1	12	rs17403620 rs17318858 rs17318955 rs17403977 rs16907989 rs7111477 rs11042883 rs11042889 rs11042892 rs16927077 rs10840444 rs1017275
MAPK1	8	rs2276008 rs9340 rs17821423 rs2298432 rs2006893 rs9607272 rs17759796 rs8141815
MAS1	1	rs220721
MDM2	3	rs937283 rs2279744 rs1470383
MICAL2	94	rs11022172 rs7111481 rs7130896 rs12803936 rs2015963 rs3763820 rs2307072 rs4756772 rs10765923 rs12577615 rs7932017 rs7942252 rs12795108 rs12790969 rs10741566 rs10765924 rs12577704 rs11022188 rs7940840 rs977244 rs2171150 rs9971381 rs901284 rs11022193 rs988189 rs4757237 rs4756775 rs11022209 rs10831742 rs7102041 rs9804570 rs1564946 rs1564947 rs923167 rs7131034 rs901302 rs2010463 rs11022214 rs10831744 rs4471395 rs7950540 rs7121956 rs7130607 rs7949360 rs6485561 rs11022242 rs10831758 rs1032151 rs17477991 rs2013262 rs3763822 rs10430830 rs12283453 rs871703 rs2279390 rs12787479 rs954428 rs11022250 rs2012580 rs2306729 rs11827638 rs12294182 rs7101833 rs10831769 rs6485587 rs7103040 rs2706643 rs2641941 rs2706645 rs1609930 rs11022257 rs2010576 rs2246778 rs3794083 rs2706637 rs4757276 rs11022262 rs2641938 rs11604904 rs2279613 rs2270511 rs12574429 rs2706627 rs1973386 rs7946327 rs1493959 rs1826608 rs11022264 rs17480838 rs7116182 rs2270513 rs3794075 rs2279616 rs8808

MYO1C	7	rs2302459 rs2302458 rs2286870 rs2302456 rs2286873 rs2286876 rs7218128
NCK1	4	rs9845460 rs1347209 rs3772388 rs1048145
NCOA4	5	rs10761581 rs10740051 rs17720205 rs41306524 rs11548236
NEDD4	15	rs11550869 rs2899593 rs17238468 rs12898589 rs8031043 rs12232351 rs2414448 rs8027843 rs10518827 rs12593446 rs12591210 rs7174459 rs12592220 rs9920283 rs16976661
NPM1	1	rs11134696
NPY2R	3	rs17376826 rs1574175 rs1047214
NRP1	53	rs1044268 rs1044210 rs2506141 rs2506143 rs2506145 rs2228638 rs2383984 rs734186 rs2474723 rs11009281 rs2474712 rs2254826 rs2269096 rs1331317 rs11009311 rs927099 rs11009313 rs12765284 rs2269091 rs12762312 rs17413155 rs17413169 rs10490939 rs1888688 rs11009323 rs2383987 rs1319013 rs11593943 rs3780869 rs10490938 rs16934292 rs11598845 rs2073320 rs4934584 rs17296436 rs17296443 rs10827227 rs10827228 rs869636 rs7079372 rs2776928 rs1331326 rs6481844 rs7910405 rs2776930 rs2776932 rs2065364 rs2804492 rs2804493 rs2776937 rs4934597 rs1360457 rs2804498
NRP2	37	rs10090 rs698909 rs849530 rs950219 rs849556 rs3771051 rs3771048 rs3771044 rs849542 rs3771038 rs861079 rs3771033 rs849523 rs849582 rs849575 rs849570 rs3771021 rs849565 rs849563 rs1996412 rs12472412 rs13026243 rs849560 rs2241155 rs3771016 rs872943 rs3771004 rs16837637 rs3771003 rs16837641 rs2241153 rs3732088 rs2160328 rs3771000 rs3770996 rs3755232 rs1990708
PALLD	104	rs11132268 rs2712135 rs2712149 rs13150330 rs2002727 rs4692943 rs10517996 rs1962363 rs10517999 rs6552861 rs10518001 rs6857497 rs7673220 rs17054290 rs11735275 rs11132283 rs9312333 rs13145788 rs1986369 rs6836618 rs6857016 rs11132322 rs17541413 rs10518011 rs10022002 rs10004025 rs1962022 rs7668720 rs17650886 rs6815330 rs17650892 rs2874112 rs17707379 rs2319909 rs4144994 rs4371580 rs4389538 rs12647503 rs2710850 rs17707568 rs2723687 rs2710851 rs2723688 rs3109799 rs2723696 rs2723698 rs12643131 rs2710828 rs2723704 rs17614077 rs10010321 rs12642267 rs17054449 rs4314247 rs12649186 rs2723705 rs13137200 rs6832582 rs17054460 rs4260495 rs4280700 rs12649675

		rs4635780 rs9884230 rs4599370 rs7697688 rs11132434 rs17542430 rs1500800 rs6852874 rs12510359 rs17542654 rs17708307 rs4692948 rs7679564 rs2247733 rs999958 rs7688994 rs17614733 rs11733873 rs1875297 rs1875296 rs7681510 rs13129779 rs6854137 rs1566499 rs6854037 rs2133911 rs867901 rs973990 rs12643033 rs1318822 rs4692552 rs7688533 rs4692553 rs13114906 rs6852229 rs2062589 rs7682426 rs867632 rs12643097 rs2047633 rs6819031 rs1500795
PCDHGB1	22	rs17097231 rs13171859 rs4151698 rs11575956 rs3806832 rs4151699 rs6867460 rs3749770 rs4912750 rs11575963 rs11958830 rs1423148 rs3805695 rs11748256 rs13361997 rs1002519 rs11952292 rs2237079 rs11744379 rs4912762 rs17286954 rs970069
PDCL3	4	rs6747613 rs2946589 rs12469806 rs2970997
PGF	2	rs8185 rs12411
PIK3R1	24	rs171648 rs7701498 rs831227 rs706713 rs13173003 rs7709243 rs12652661 rs173704 rs173702 rs4122269 rs1823023 rs173703 rs6893676 rs34303 rs863818 rs34309 rs2302975 rs3730082 rs6876003 rs3815701 rs34306 rs1550805 rs831125 rs3730089
PLCG1	4	rs2866370 rs753381 rs6072299 rs4297946
PLVAP	4	rs4808078 rs7252581 rs16981755 rs10417806
PLXNA1	7	rs6764158 rs732737 rs747967 rs9289290 rs4679325 rs9851451 rs3749395
PPM1A	3	rs7155841 rs10142834 rs12434739
PPM1B	4	rs1453863 rs17039151 rs4952703 rs2053456
PRRG4	7	kgp11715177 rs33962176 rs11605633 rs7944652 rs11032017 kgp8085505 rs7933966
PTGER3	49	rs959 rs1327460 rs6656853 rs6672081 rs7530738 rs7533733 rs6685546 rs6685646 rs17481440 rs1536537 rs1536261 rs1576055 rs4649932 rs35702222 rs1409166 rs1409165 rs1327464 rs1409162 rs4420040 rs7530658 rs2182325 rs11209714 rs875727 rs17541722 rs7539384 rs17542063 rs6424410 rs7538034 rs6670616 rs12067140 rs510414 rs475468 rs1409984 rs1071020 rs571705 rs977214

		rs2072947 rs479934 rs2206343 rs2268055 rs2300168 rs5693 rs5691 rs1022528 rs8179390 rs2300179 rs10889906 rs2050065 rs2817867
PTPN11	4	rs11066301 rs17822304 rs12423190 rs11066323
PTPN12	8	rs9886084 rs10808113 rs2286894 rs1024723 rs7776973 rs17381884 rs17467232 kgp4610958
PTPN6	5	rs2301262 rs10774452 rs2110071 rs2071079 rs759052
PTPRB	42	rs431716 rs630608 rs17226367 rs2278346 rs17226374 rs2567142 rs919594 rs2567140 rs11178281 rs3782377 rs2567137 rs2584026 rs2567133 rs2116209 rs12314266 rs4761222 rs2303963 rs2717440 rs7954837 rs991833 rs2116211 rs2034011 rs2304821 rs2717418 rs2584011 rs2165627 rs11178317 rs2465811 rs11178321 rs2717430 rs2583999 rs751363 rs2439732 rs2465810 rs10506598 rs7298147 rs2717425 rs11178333 rs17814416 rs17108441 rs1442205 rs2717417
PTPRR	47	rs2717445 rs10879175 rs7298378 rs7314925 rs11178364 rs12580224 rs2089975 rs1398602 rs6581958 rs1156461 rs11178376 rs12813125 rs972769 rs1398599 rs11178388 rs3803036 rs7974346 rs4760933 rs6581964 rs1513098 rs7968934 rs7297717 rs7306190 rs4760744 rs4760810 rs10879198 rs1022242 rs17814482 rs2048607 rs7956670 rs2203232 rs17108861 rs10784870 rs12229663 rs4294640 rs12305560 rs12297391 rs10879213 rs17108998 rs3923909 rs10879214 rs11178478 rs4760847 rs3924187 rs6581971 rs7965899 rs4595639
PTPRZ1	22	rs740965 rs1007784 rs12669706 rs1019221 rs960930 rs6466808 rs6970897 rs2690271 rs1196510 rs13246377 rs1209633 rs3817483 rs1196505 rs2693657 rs1196473 kgp11436861 rs1147504 rs1147498 rs1147492 rs1147491 rs1147489 rs1147487
RAB5A	9	rs11128928 rs4858660 rs17181547 rs4241539 rs2127956 rs4398451 rs9835991 rs13085694 rs8682
RASA1	5	rs6452750 rs35148638 rs10045850 rs2923742 rs10057748
RNF123	4	rs11130216 rs1491985 kgp9864706 rs11130218
SCGB2A2	1	rs17709552

SCUBE2	31	rs1136966 rs1367 rs2056902 rs3751057 rs7109896 rs3794149 rs1883100 rs10743098 rs1883099 rs10840164 rs6486112 rs3751055 rs10769988 rs7106593 rs3751051 rs3763904 rs7112378 rs7130913 rs6486125 rs2003906 rs4910431 rs7107892 rs11606516 rs7929797 rs2647528 rs1121629 rs11042182 rs3898554 rs4910443 rs10769990 rs10769992
SERPINF1	5	rs11658342 rs1136287 rs12603825 rs8074840 rs6828
SH2D2A	2	rs926103 rs2150906
SHB	12	rs776023 rs776022 rs776015 rs735740 rs3827519 rs3802414 rs10973635 rs12345885 rs7047051 rs7856790 rs943936 rs4878743
SHC2	11	rs8902 rs1046822 rs16990450 rs10426188 rs12981152 rs10408164 rs8112380 kgp471423 rs10409912 rs4919871 rs740871
SP1	2	rs3741651 rs17695156
SP3	3	rs6711060 rs4508563 rs10190140
SPARC	8	rs707156 rs3210714 rs729853 rs725937 rs2881558 rs17718347 rs11745387 rs17112187
SRC	13	rs7275012 rs16986606 rs6017996 rs6018027 rs6063022 rs6018088 rs6090575 rs12329503 rs6090585 rs754625 rs6018257 rs1570209 rs17785475
SRPK1	3	rs17704843 rs3761981 rs11968721
STAT3	6	rs1053005 rs1053004 rs8069645 rs6503695 rs744166 rs4796791
STYX	3	rs10483617 rs11625099 rs10873061
TFAP2A	6	rs537112 rs533558 rs303050 rs3798696 rs1675414 rs303055
TMEM52B	8	kgp11971666 rs7315498 rs7305138 rs10505752 rs12313003 rs17808107 rs4764306 rs4764308
TNF	1	rs3093662

TNFRSF25	1	rs11800462
TP53	7	rs8073498 rs12949853 rs1614984 rs1625895 rs1042522 rs8079544 rs11652704
TRIM68	2	rs3750992 rs931811
U2AF1	3	rs3788054 rs4920039 rs1789956
UBC	2	rs41276688 rs13624
USF2	3	rs2515622 kgp22836814 rs10405246
VCL	10	rs12250729 rs4746166 rs10458640 rs10458657 rs11000851 rs11000864 rs11000869 rs767809 rs2279648 rs3793921
VEGFA	7	rs25648 rs833068 rs833070 rs3024994 rs3025010 rs3025039 rs3025053
VEGFB	2	rs11603042 rs4930152
VEGFC	8	rs2877961 rs17697359 rs1485762 rs1485766 rs11947611 rs3775195 rs2171083 rs4557213
VHL	1	rs1642742
VPS35	1	rs700582
VTN	3	rs2277667 kgp4183944 rs2071379
YWHAG	2	rs2908191 rs917424
ZCCHC6	4	rs7035034 rs4587414 rs10115526 rs700759
ZSCAN21	2	rs11558475 rs12705070

Genes and SNPs used in the Part 2 (VEGF interaction network) analyses. Genes are listed in alphabetical order.

LD pruning: PLINK was used for genotype extraction, followed by LD-based pruning (using the PLINK the command *indep-pairwise* with a window of 50 SNPs, a step size of 5 SNPs (Génin et al., 2011; Pearson et al., 2011) and a threshold of 0.8 (LD > 0.8 removed) was used.

2 RESULTS

Table S6: Results of the 1-way Cox-MDR runs (n=20) examining the MMP gene SNPs (n=201).

Run #	CVC	Testing Balance Accuracy	Best SNP	Genotype Risk Categorization (High Risk Shown)
10	2	0.512963	rs11225388	0:230
12	4	0.590351	rs11225388	0:230
17	4	0.595105	rs11225388	0:230
6	3	0.595118	rs11225388	0:230
5	5	0.596491	rs11225388	0:230
13	3	0.597114	rs11225388	0:230
4	4	0.598295	rs11225388	0:230
3	3	0.610472	rs11225388	0:230
1	3	0.613158	rs11225388	0:230
7	4	0.616848	rs11225388	0:230
16	4	0.617092	rs11225388	0:230
19	4	0.62069	rs11225388	0:230
11	4	0.62193	rs11225388	0:230
20	5	0.630688	rs11225388	0:230
8	4	0.642281	rs11225388	0:230
2	4	0.643003	rs11225388	0:230
9	4	0.64537	rs11225388	0:230
15	4	0.648148	rs11225388	0:230
18	4	0.649482	rs11225388	0:230
**14**	**5**	**0.656477**	**rs11225388**	**0:230**

CVC: Cross-Validation Consistency. Using our selection procedure, rs11225388, with AA genotype being the high risk genotype, AG and GG genotypes being the low risk genotypes, was found to be the most frequent (and top) MDR model. Genotype risk classification format: SNP Genotype: Number of patients in genotype risk category. Genotypes are presented with additive coding (0=major allele homozygous genotype; 1=heterozygous genotype, 2=minor allele homozygous). The top model is bolded. Data in this table is sorted by TBA and genotype risk categorization.

Figure S2. Kaplan Meier curves of the models identified by GMDR 0.9 in MMP SNP interaction analysis

A. 2-way model:

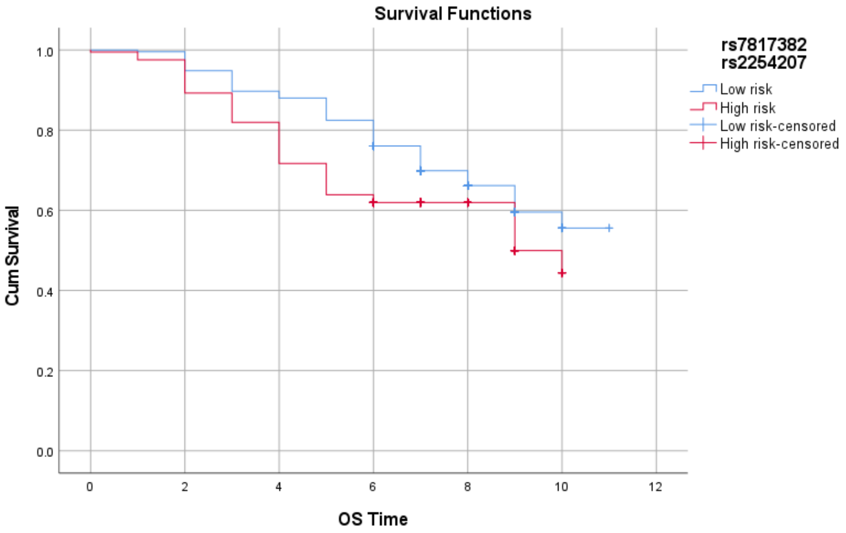

Log-rank p= 0.0152154116604927
Red: (AA,CA),(AA,CC),(GA,AA),(GA,CC),(GG,CA)
Blue: All other genotype combinations

B. 3-way model:

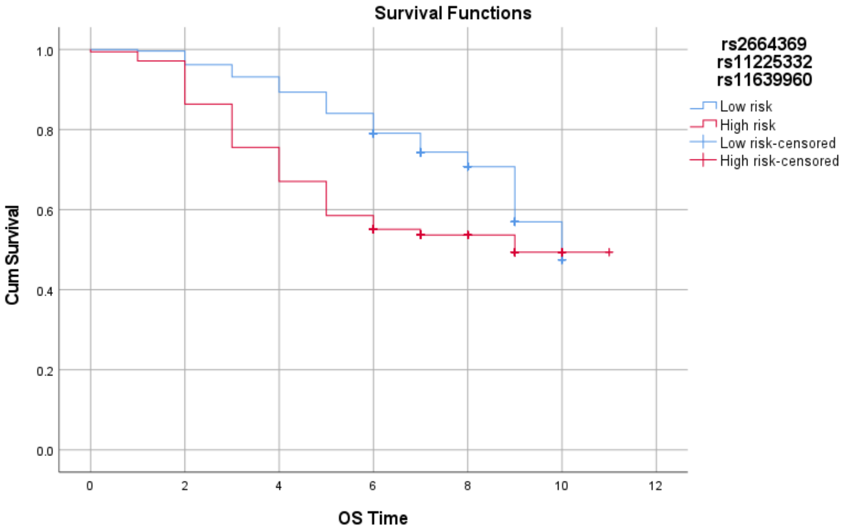

Log-rank p = 0.0000209959191927817
Red: (0TT,0TT,2GG), (0TT,1CT,1GA), (0TT,1CT,2GG), (0TT,2CC,1GA), (1GT,0TT,0AA), (1GT,0TT,1GA), (1GT,1CT,2GG), (1GT,2CC,2GG), (2GG,0TT,0AA), (2GG,1CT,2GG), (2GG,2CC,0AA), (2GG,2CC,2GG)
Blue: All other genotype combinations

Figure S3. Kaplan-Meier curves for models identified in the VEGF interaction network analysis by GMDR 0.9.

<u>Red: high risk genotypes, blue: low risk genotypes</u>

VEGFA

1-way model, iteration 1:

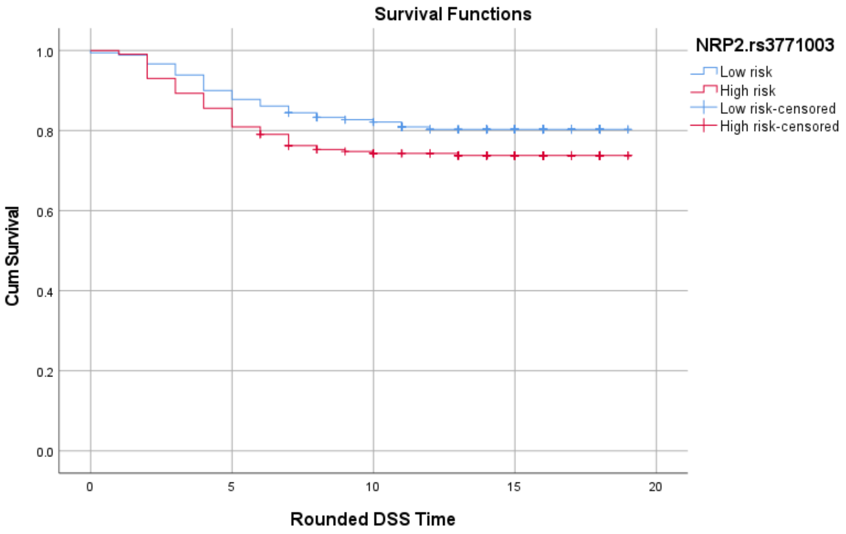

Log-rank p = 0.1064349793977
Red: GG and TT
Blue: TG

1-way model, iteration 2:

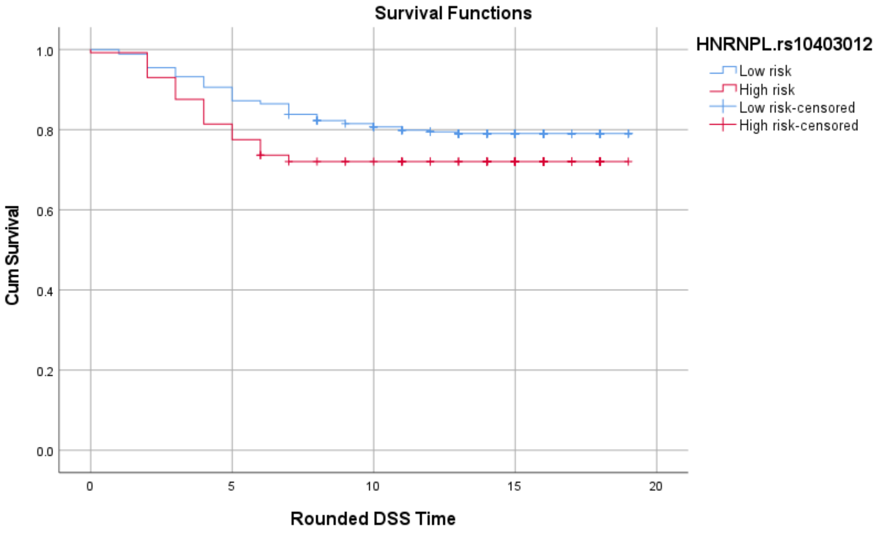

Log-rank p = 0.0749006184227615
Red: AA
Blue: GA, GG

2-way model:

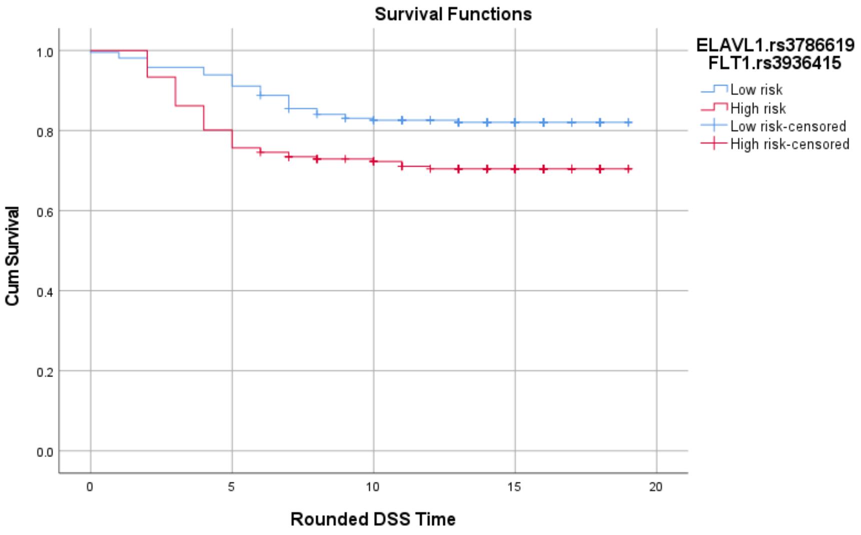

Log-rank p = 0.00393290069799821
Red: (GG,AA), (AG,GG), (AA,GG), (AA,AG)
Blue: All other genotype combinations

3-way model:

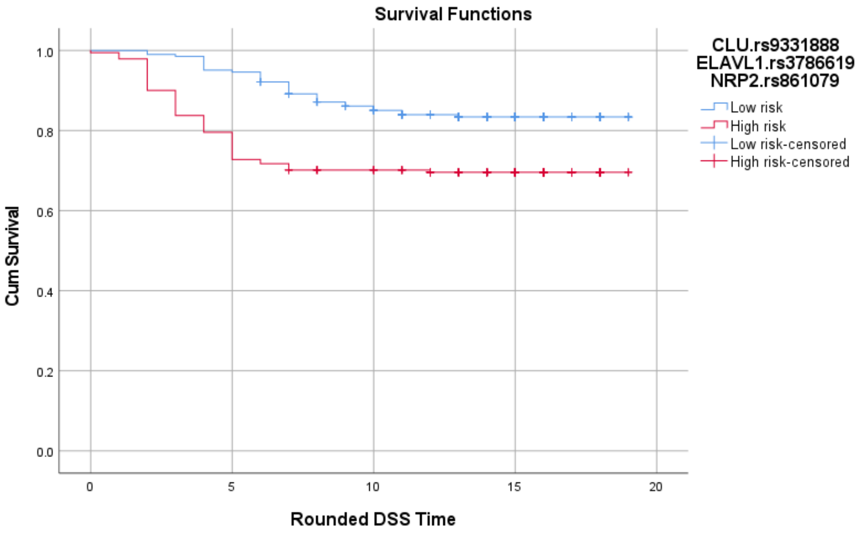

Log-rank p = 0.000238697896697943
Red: (GG,GG,TC), (GG,GG,TT), (GG,AG,TC), (GG,AA,CC), (GG,AA,TT), (CG,AG,CC), (CG,AG,TT), (CG,AA,CC), (CC,GG,TC), (CC,GG,TT), (CC,AG,TT), (CC,AA,TT)
Blue: All other genotype combinations

VEGFB

1-way model:

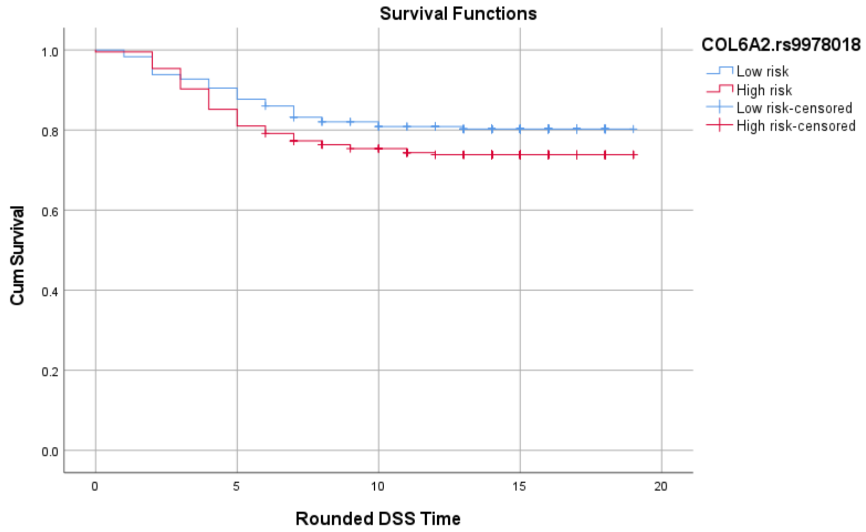

Log-rank p = 0.133385031290691
Red: AA and GG
Blue: GA

2-way model:

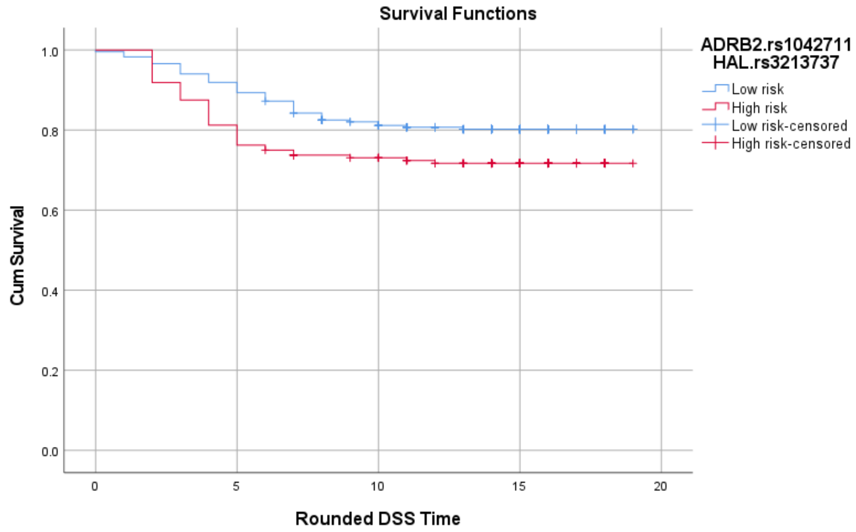

Log-rank p = 0.0309353056998482
Red: (TT,CT), (CT,TT), (CC,CT)
Blue: All other genotype combinations

VEGFC

2-way model:

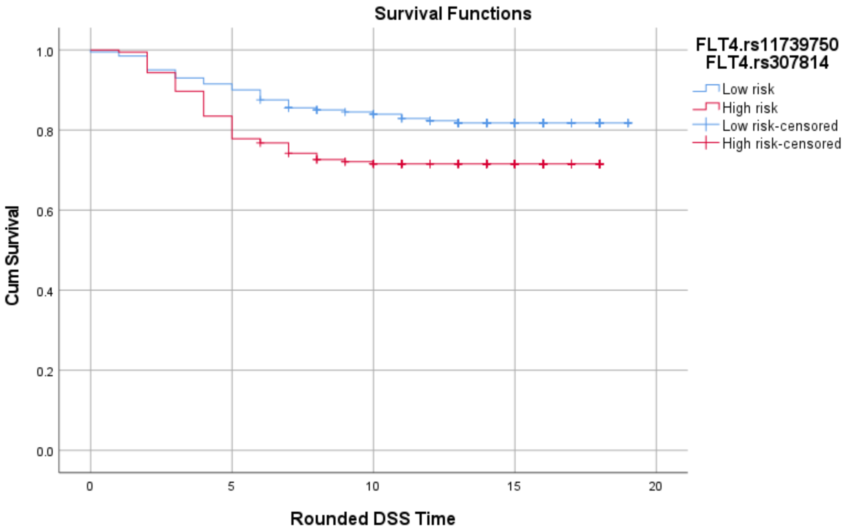

Log-rank p = 0.0126227526320323
Red: (CC,TC), (TC,CC), (TC,TT), (TT,TC)
Blue: All other genotypes except (TT, TT)

3-way model:

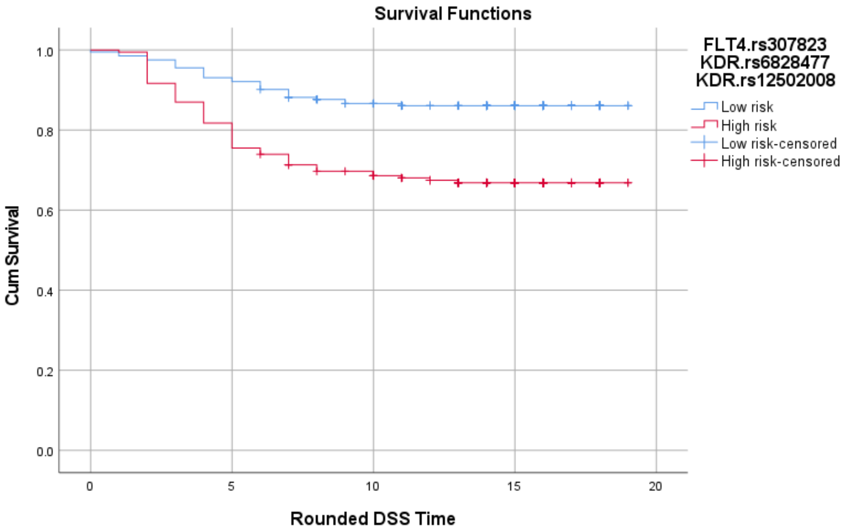

Log-rank p = 5.89522330356229E-06
Red: (AA,TT,TG), (AA,CT,GG), (AA,CC,GG), (AA,CC,TG), (GA,TT,GG), (GA,CT,TG), (GA,CC,TT), (GG,TT,TG), (GG,CT,TG), (GG,CT,TT)
Blue: All other genotype combinations except (GG,CC,TG) and (GG,CC,TT)

VEGFR1

1-way model:

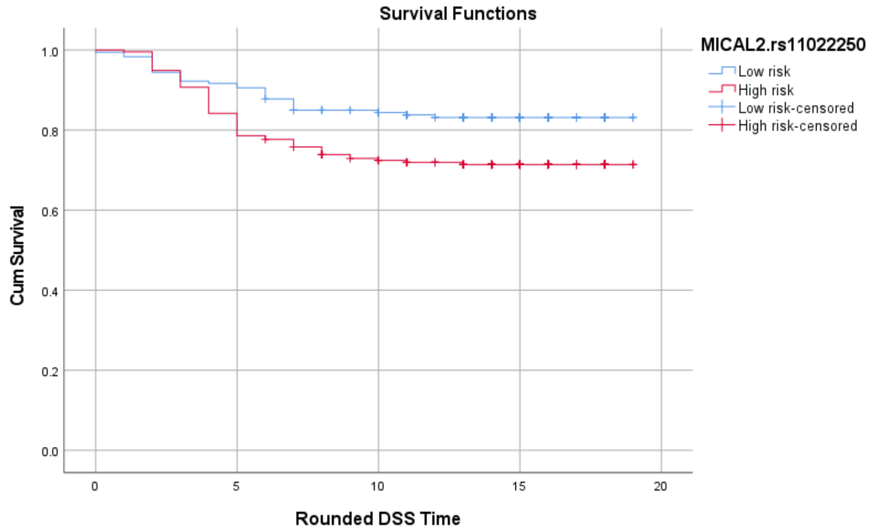

Log-rank p = 0.00694530789778492
Red: TT
Blue: GT, GG

2-way model:

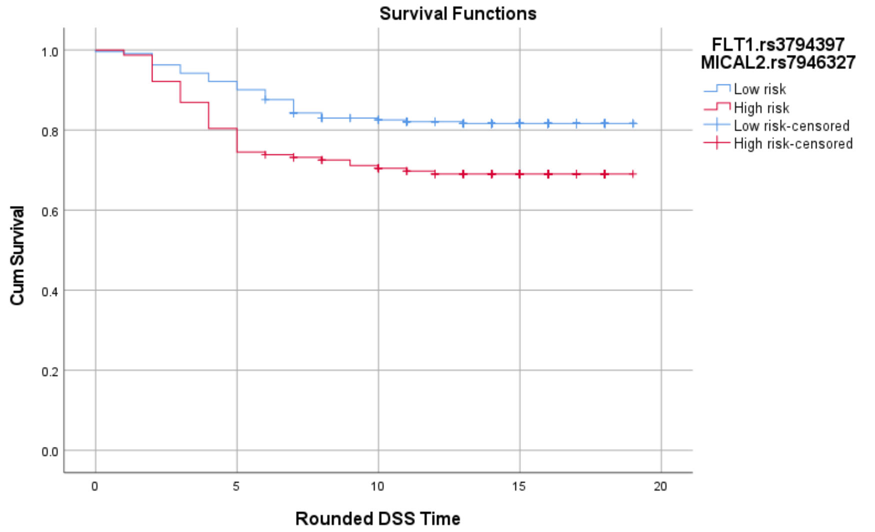

Log-rank p = 0.00244383578765834
Red: (CC,AA), (TC,CA), (TT,CA)
Blue: All other genotype combinations

3-way model:

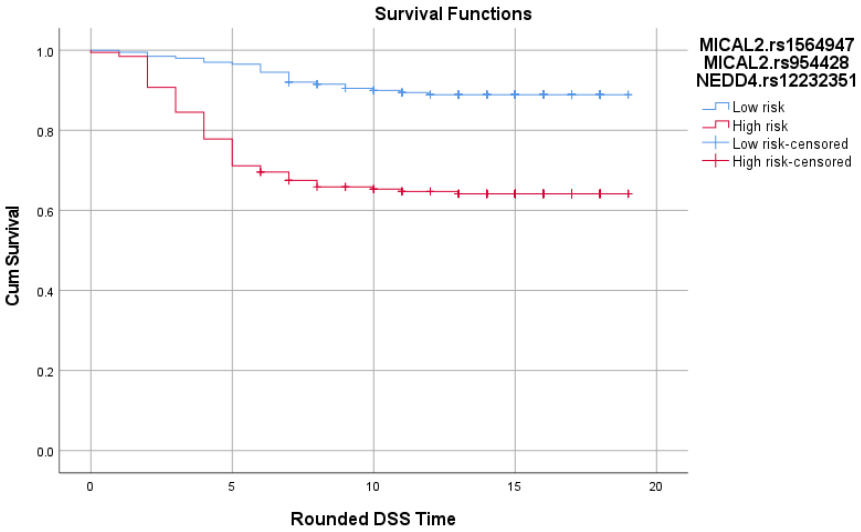

Log-rank p = 1.50474809470499E-09
Red: (GG,AA,TT), (GG,AA,AA), (GG,GA,TT), (GG,GA,AT), (GG,GG,TT), (AG,AA,TT), (AG,GA,AT), (AA,GG,TT), (AA,GG,AA)
Blue: All other genotype combinations except (AA,AA,TT) and (AA,AA,AA)

VEGFR2

1-way model, iteration 1:

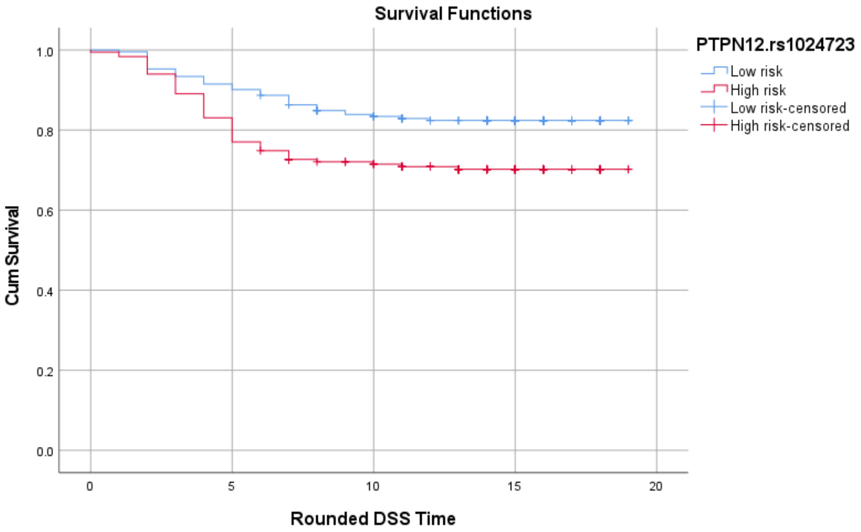

Log-rank p = 0.00325375804427782
Red: TT and CC
Blue: TC

1-way model, iteration 2:

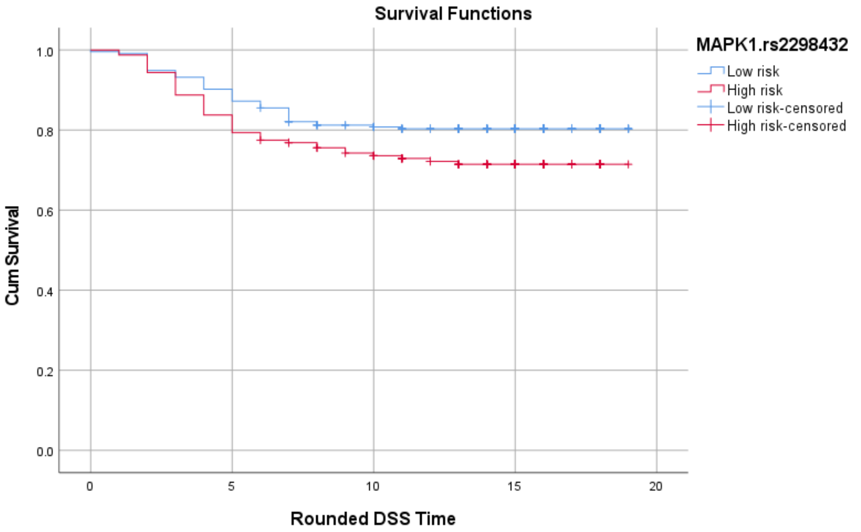

Log-rank p = 0.0440341835682624
Red: CC
Blue: AC, AA

2-way model:

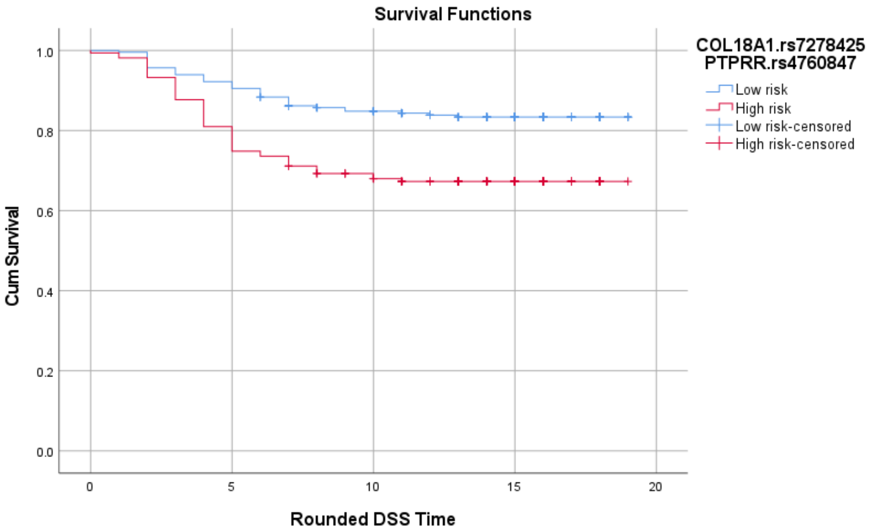

Log-rank p = 0.000128426652769784
Red: (CC,GA), (TC,AA)
Blue: All other genotype combinations

3-way model:

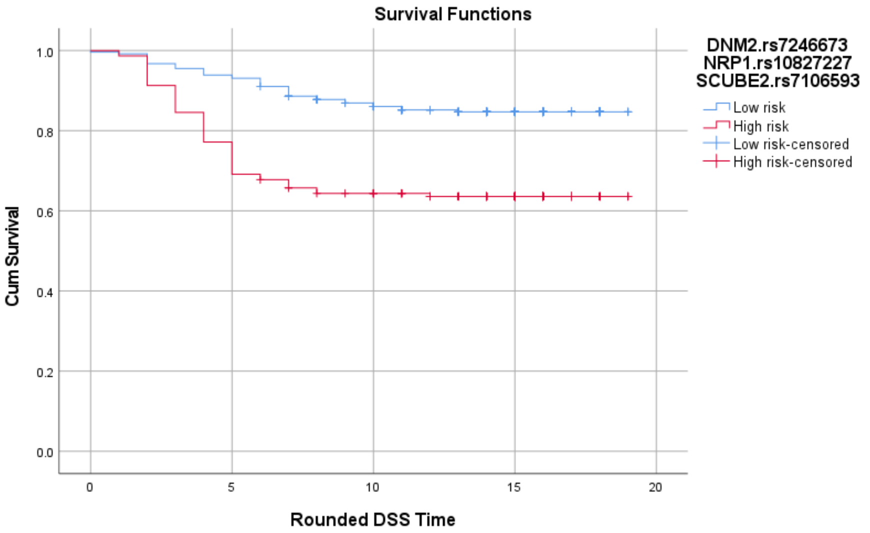

Log-rank p = 3.03572318253623E-07
Red: (TG,CC,GT), (TG,TC,TT), (TG,TC,GG), (TG,TT,TT), (TG,TT,GT), (TG,TT,GG), (TT,CC,GG), (TT,TC,GT), (TT,TC,GG), (TT,TT,GT)
Blue: All other genotype combinations

VEGFR3

1-way model, iteration 1:

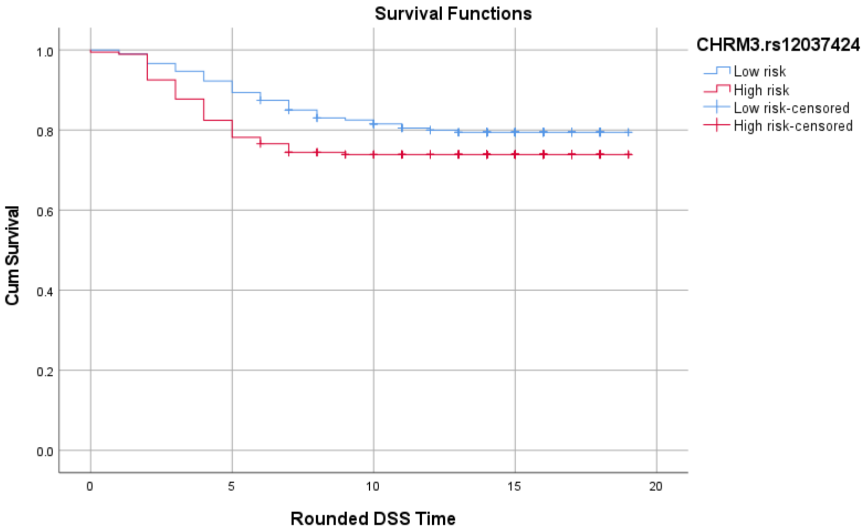

Log-rank p = 0.109869998601945
Red: TT
Blue: CT, CC

1-way model, iteration 2:

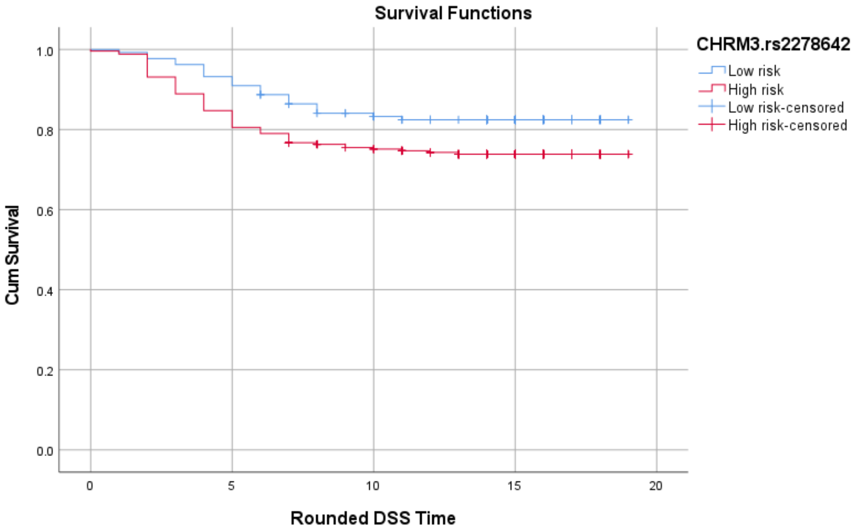

Log-rank p = 0.0443756555088326
Red: TG, TT
Blue: GG

1-way model, iteration 3:

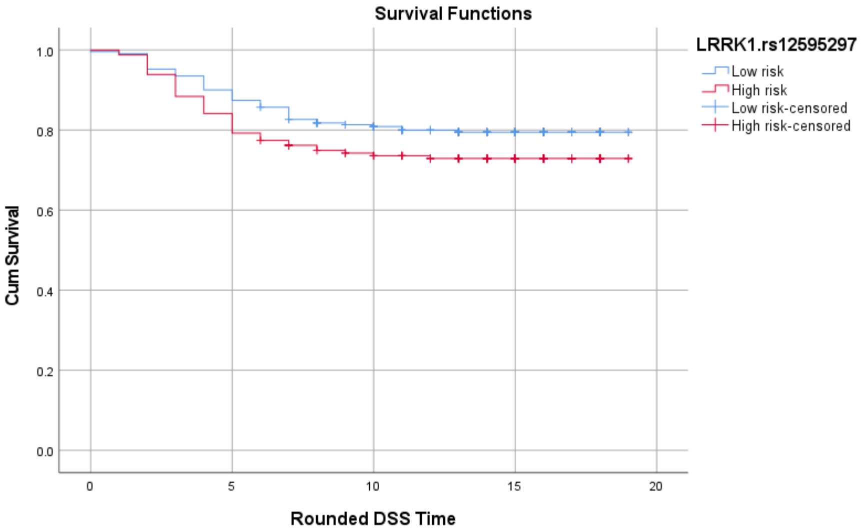

Log-rank p = 0.105405395808711
Red: GT
Blue: TT, GG

1-way model, iteration 4:

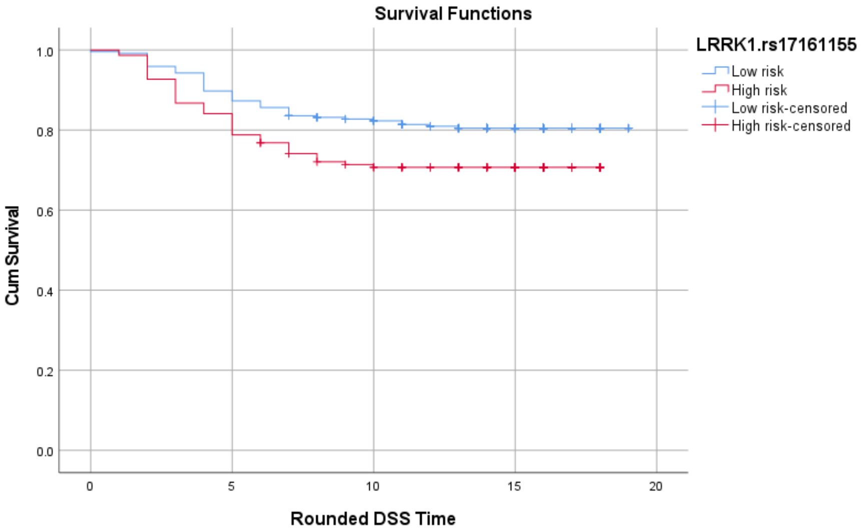

Log-rank p = 0.0190727872695036
Red: GG
Blue: AG, AA

2-way model:

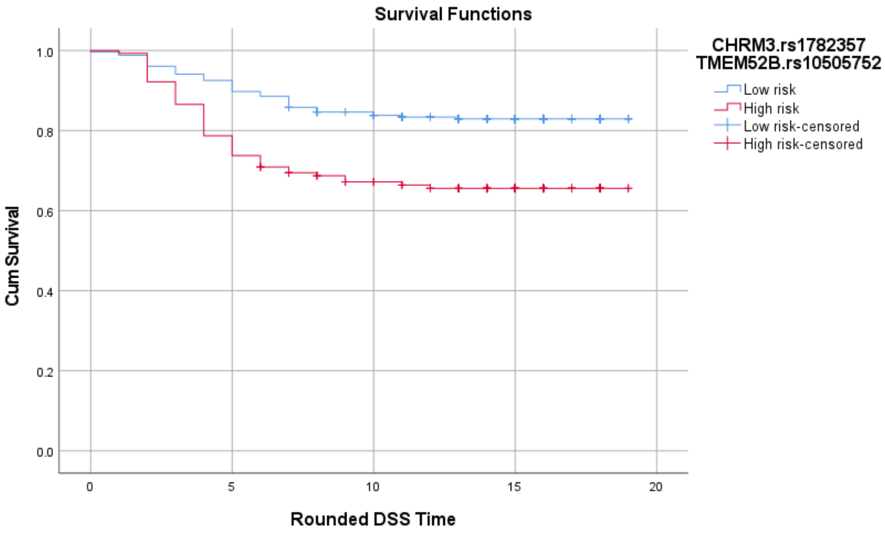

Log-rank p = 0.000050799778711075
Red: (CC,CC), (TC,TC), (TC,TT), (TT,TT)
Blue: All other genotype combinations

3-way model:

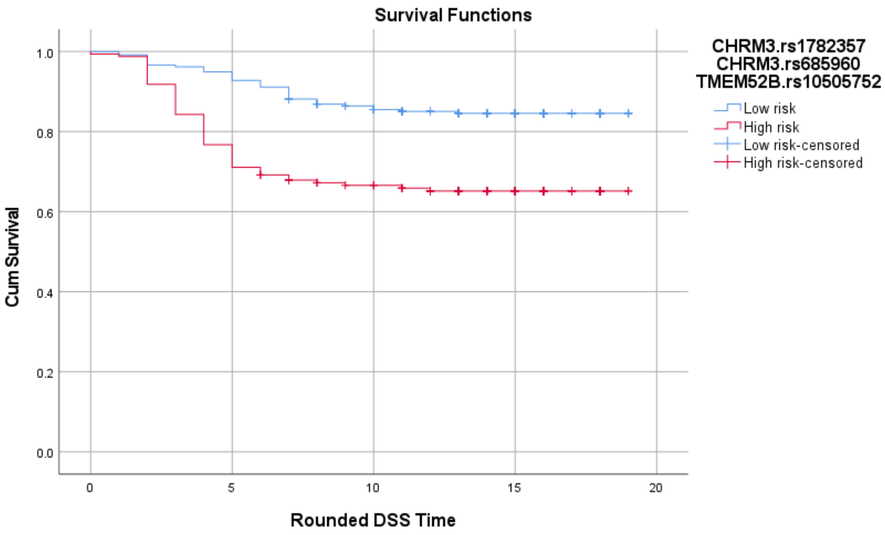

Log-rank p = 0.0000018569215896555
Red: (CC,TT,CC), (CC,CT,CC), (TC,TT,TC), (TC,TT,TT), (TC,CT,CC), (TT,TT,TT), (TT,CT,CC), (TT,CT,TT)
Blue: All other genotypes except (CC,CC,CC), (CC,CC,TC), (CC,CC,TT), (TC,CC,CC), (TC,CC,TC), (TC,CC,TT), (TT,CC,CC), and (TT,CC,TT)

PIGF

1-way model:

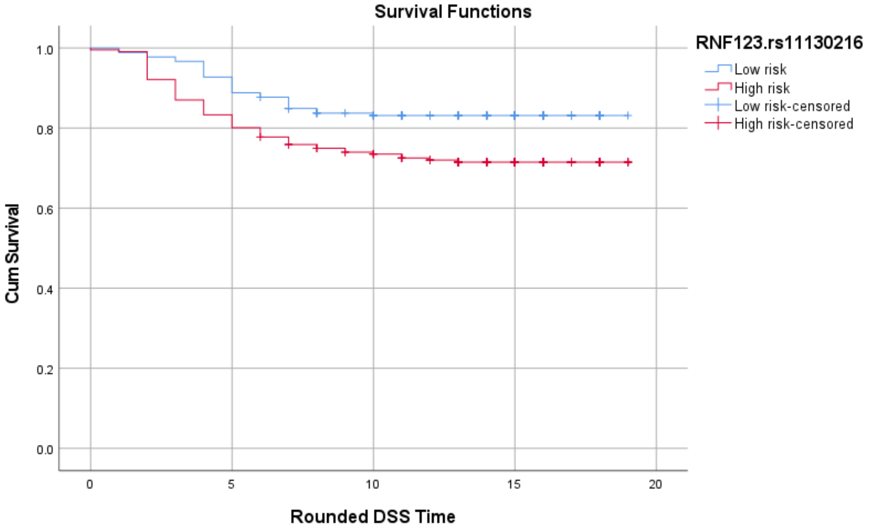

Log-rank p = 0.00581230466407173
Red: AC, AA
Blue: CC

3-way model:

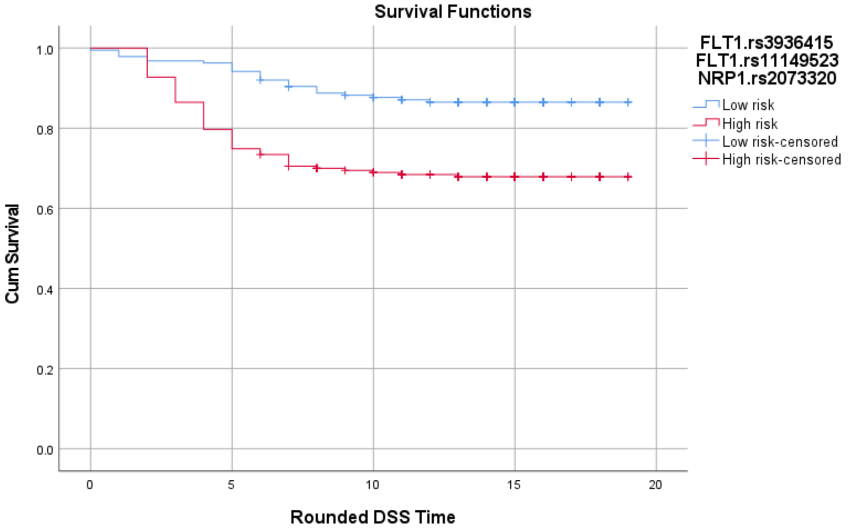

Log-rank p = 6.66585629309672E-06
Red: (GG,GG,CC), (GG,GG,TC), (GG,AG,CC), (GG,AG,TT), (GG,AA,CC), (GG,AA,TT), (AG,GG,TT), (AG,AG,TC), (AG,AA,TC), (AG,AA,TT), (AA,GG,CC), (AA,AA,CC)
Blue: All other genotype combinations

Table S7. SNPs annotated as eQTLs and identified by either Cox-MDR or GMDR 0.9 in this study.

**Interactor set**	**SNP**	**Gene**	**MAF**	**RegulomeDB rank/score**	**RegulomeDB if eQTL - which tissue?**	**RegulomeDB if eQTL - which gene?**	**GTEx (if eQTL?) in transverse colon**	**GTEx if eQTL in transverse colon - which gene?**	**GTEx (if eQTL?) in sigmoid colon**	**GTEx (if eQTL?) in sigmoid colon - which gene?**
	**PART 1 (MMP family genes)**									
	**2-way GMDR 0.9**									
	rs7817382 and rs2254207									
	rs7817382	MMP16	0.2506	6	No results	NA	cis-eQTL	MMP16 (minor allele G - lower expression)	No results	NA
	rs2254207	MMP24	0.2585	4	No results	NA	No results	NA	cis-eQTL	MMP24-AS1 (minor allele C - lower expression)
	**3-way GMDR 0.9**									
	rs2664369, rs11225332 and rs11639960									

	rs11639960	MMP2	0.3497	1f	monocyte (cis-eQTL)	AYTL1 (also named LPCAT2, (based on:(Zeller et al., 2010) minor allele G - lower expression)	No results	NA	No results	NA
	**PART 2 (VEGF family networks)**									
	**1-way Cox-MDR**									

PIGF [also identified by GMDR 0.9 GMDR]	rs11130216	RNF123	0.3125	4	No results	NA	cis-eQTL	RBM6 (minor allele A -lower expression),<br><br>UBA7 (minor allele A -lower expression),<br><br>GPX1 (minor allele A -lower expression),<br><br>AMT (minor allele A - higher expression)	cis-eQTL	RBM6 (minor allele A - lower expression),<br><br>AMT (minor allele A - higher expression),<br><br>CCDC36 (minor allele A - higher expression),<br><br>MST1R (minor allele A - higher expression),<br><br>CDHR4 (minor allele A - higher expression)
	**1-way GMDR 0.9**									
VEGFR2 (iteration 1)	rs1024723	PTPN12	0.41	5	No results	NA	cis-eQTL	APTR (minor allele T - higher expression)	cis-eQTL	APTR (minor allele T - higher expression)

PIGF	rs11130216 (iteration 1)	RNF123	0.3125	4	No results	NA	cis-eQTL	RBM6 (minor alleles A - lower expression),<br><br>UBA7 (minor alleles A - lower expression),<br><br>GPX1 (minor alleles A - lower expression),<br><br>AMT (minor alleles A - higher expression)	cis-eQTL	RBM6 (minor allele A - lower expression),<br><br>AMT (minor allele A - higher expression),<br><br>CCDC36 (minor allele A - higher expression),<br><br>MST1R (minor allele A - higher expression),<br><br>CDHR4 (minor allele A - higher expression)
VEGFR2	rs2298432 (iteration 2)	MAPK1	0.3663	3a	No results	NA	cis-eQTL	LL22NC03-86G7.1 (minor allele A - higher expression),<br><br>TOP3BP1 (minor allele A - lower expression)	cis-eQTL	LL22NC03-86G7.1 (minor allele A - higher expression),<br><br>TOP3BP1 (minor allele A - lower expression),<br><br>PPIL2 (minor allele A - higher expression)

VEGFR3	rs17161155 (iteration 4)	LRRK1	0.3887	1f	monocyte (cis-eQTL)	LRRK1 (based on (Zeller et al., 2010), minor allele A - lower expression)	No results	NA	No results	NA
	**2-way GMDR 0.9**									
VEGFA	ELAVL1.rs3786619 FLT1.rs3936415									
	rs3786619	ELAVL1	0.47	4	No results	NA	cis-eQTL	CTD-3193O13.8 (minor allele A - lower expression)	cis-eQTL	CTD-3193O13.8 (minor allele A - lower expression),<br><br>CTD-2325M2.1 (minor allele A - higher expression)
VEGFB	ADRB2.rs1042711 HAL.rs3213737									
	rs3213737	HAL	0.4088	5	No results	NA	cis-eQTL	AMDHD1 (minor allele A - lower expression)	cis-eQTL	AMDHD1 (minor allele A - lower expression)

VEGFC	FLT4.rs11739750<br>FLT4.rs307814									
	rs11739750	FLT4	0.2175	1f	monocyte (cis-eQTL)	SCGB3A1 (based on(Zeller et al., 2010): minor allele T - higher expression)	No results	NA	No results	NA
	**3-way GMDR 0.9**									
VEGFA	CLU.rs9331888<br>ELAVL1.rs3786619<br>NRP2.rs861079									
	rs3786619	ELAVL1	0.47	4	No results	NA	cis-eQTL	CTD-3193O13.8 (minor allele A - lower expression)	cis-eQTL	CTD-3193O13.8 (minor allele A - lower expression),<br><br>CTD-2325M2.1 (minor allele A - higher expression)

VEGFB	ADRB2.rs1042711<br>NRP1.rs17296436<br>VEGFB.rs11603042									
	rs11603042	VEGFB	0.36	5	No results	NA	cis-eQTL	TRPT1 (minor allele T - higher expression),<br><br>FKBP2 (minor allele T - higher expression)	cis-eQTL	TRPT1 (minor allele T - higher expression),<br><br>FKBP2 (minor allele T - higher expression)
VEGFC	FLT4.rs307823<br>KDR.rs6828477<br>KDR.rs12502008									
	rs12502008	KDR	0.3613	4	No results	NA	No results	NA	cis-eQTL	SRD5A3 (minor allele T - higher expression)
VEGFR1	MICAL2.rs1564947<br>MICAL2.rs954428<br>NEDD4.rs12232351									

	rs12232351	NEDD4	0.335	6	No results	NA	cis-eQTL	NEDD4 (minor allele A - higher expression)	No results	NA
VEGFR2	DNM2.rs7246673 NRP1.rs10827227 SCUBE2.rs7106593									
	rs7106593	SCUBE2	0.425	7	No results	NA	No results	NA	cis-eQTL	TRIM66 (minor allele G - lower expression),<br><br>SCUBE2 (minor allele G - higher expression)

Only the variants that are annotated as an eQTL are shown in this table. eQTL: expression quantitative trait locus; MAF: Minor Allele Frequency; NA: not applicable

References

BioGRID | Database of protein, chemical, and genetic interactions. https://thebiogrid.org/ [Accessed January 29, 2020].

Génin, E., Schumacher, M., Roujeau, J.-C., Naldi, L., Liss, Y., Kazma, R., et al. (2011). Genome-wide association study of Stevens-Johnson Syndrome and Toxic Epidermal Necrolysis in Europe. Orphanet J. Rare Dis. 6, 52.

Kent WJ, Sugnet CW, Furey TS, Roskin KM, Pringle TH, Zahler AM, Haussler D (2002). The human genome browser at UCSC. Genome Res. 12, 996–1006.

Microsoft R Open (2019). Microsoft Corporation. https://mran.microsoft.com/open [Accessed March 22, 2022]

Oughtred, R., Rust, J., Chang, C., Breitkreutz, B.-J., Stark, C., Willems, A., et al. (2021). The BioGRID database: A comprehensive biomedical resource of curated protein, genetic, and chemical interactions. Protein Sci. 30, 187–200.

Pearson, J. P., Williams, N. M., Majounie, E., Waite, A., Stott, J., Newsway, V., et al. (2011). Familial frontotemporal dementia with amyotrophic lateral sclerosis and a shared haplotype on chromosome 9p. J. Neurol. 258, 647–655.

R Core Team (2017). R: a language and environment for statistical computing. Vienna, Austria: R Foundation for Statistical Computing https://www.R-project.org/.

Ritchie, M. D., Hahn, L. W., Roodi, N., Bailey, L. R., Dupont, W. D., Parl, F. F., et al. (2001). Multifactor-dimensionality reduction reveals high-order interactions among estrogen-metabolism genes in sporadic breast cancer. Am. J. Hum. Genet. 69, 138–147.

RStudio Team (2015). RStudio: Integrated Development Environment for R. Boston, MA: RStudio, Inc. http://www.rstudio.com/.

Stark, C., Breitkreutz, B.-J., Reguly, T., Boucher, L., Breitkreutz, A., and Tyers, M. (2006). BioGRID: a general repository for interaction datasets. Nucleic Acids Res. 34, D535-539.

Yates, B., Braschi, B., Gray, K. A., Seal, R. L., Tweedie, S., and Bruford, E. A. (2017). Genenames.org: the HGNC and VGNC resources in 2017. Nucleic Acids Res. 45, D619–D625.

Zeller, T., Wild, P., Szymczak, S., Rotival, M., Schillert, A., Castagne, R., et al. (2010). Genetics and beyond--the transcriptome of human monocytes and disease susceptibility. PLoS One 5, e10693.
